# Supplementary material for: Long COVID cognitive sequelae 6 months postinfection and beyond: a scoping review protocol
Source: BMJ Open. 2024 Jun 6;14(6):e084798. doi: 10.1136/bmjopen-2024-084798 (PMC11163643; doi:10.1136/bmjopen-2024-084798)
Supplement: Author's manuscript [file bmjopen-2024-084798.draft_revisions.pdf]

BMJ Open is committed to open peer review. As part of this commitment we make the peer review history of every article we publish publicly available.

When an article is published we post the peer reviewers' comments and the authors' responses online. We also post the versions of the paper that were used during peer review. These are the versions that the peer review comments apply to.

The versions of the paper that follow are the versions that were submitted during the peer review process. They are not the versions of record or the final published versions. They should not be cited or distributed as the published version of this manuscript.

BMJ Open is an open access journal and the full, final, typeset and author-corrected version of record of the manuscript is available on our site with no access controls, subscription charges or pay-per-view fees (http://bmjopen.bmj.com).

If you have any questions on BMJ Open's open peer review process please email info.bmjopen@bmj.com

BMJ Open

Long COVID Cognitive Sequelae 6 Months Post-Infection and Beyond: A Scoping Review Protocol

Journal:	*BMJ Open*
Manuscript ID	bmjopen-2024-084798
Article Type:	Protocol
Date Submitted by the Author:	28-Jan-2024
Complete List of Authors:	Monteiro, Sara; Luxembourg Institute of Health, Department of Precision Health<br>Dessenne, Coralie; Luxembourg Institute of Health, Science Office<br>Perquin, Magali ; Luxembourg Institute of Health, Department of Precision Health
Keywords:	Post-Acute COVID-19 Syndrome, Psychometrics, Infectious disease/HIV < NEUROLOGY, Chronic Disease

SCHOLARONE™
Manuscripts

Long COVID Cognitive Sequelae 6 Months Post-Infection and Beyond:

A Scoping Review Protocol

Sara Monteiro[1,*], Coralie Dessenne[2], Magali Perquin[1].

[1] Department of Precision Health, Luxembourg Institute of Health, 1A-B, rue Thomas Edison, 1445 Strassen, Luxembourg

[2] Science Office, Luxembourg Institute of Health, 1A-B, rue Thomas Edison, 1445 Strassen, Luxembourg

***Corresponding Author:**

Sara Monteiro: sara.monteiro@lih.lu, ORCID: 0000-0001-9145-0063

Author Note:

Coralie Dessenne: coralie.dessenne@lih.lu, ORCID: 0000-0001-7723-0304

Magali Perquin: magali.perquin@lih.lu, ORCID: 0000-0002-0386-0212

Declaration: No conflict of interest to declare.

For peer review only - http://bmjopen.bmj.com/site/about/guidelines.xhtml

Abstract

Introduction: The novel and expanding field of long COVID research has undergone diverse methodological approaches in recent years. This protocol lays out the research methodology, which aims at identifying nuances in present research and underscores the necessity for a more precise understanding of prolonged cognitive sequelae and their relation to initial disease severity. The findings will add valuable insights for the development of targeted rehabilitation and healthcare interventions and thereby aid patients, clinicians, policymakers, and researchers.

Methods and Analysis: To map current research in the field, a scoping review will be conducted and documented in accordance with the Preferred Reporting Items for Systematic Reviews and Meta-Analyses - Scoping Review Extension standards. A systematic search of scientific databases (PubMed, EMBASE), presented 1409 eligible results, published up to 21 December 2023. After removal of duplicates, 997 articles were extracted for screening. Two independent reviewers will screen for titles, abstracts and full texts, to chart extracted data using charting software. Data for various variables, such as journal info, studied population demographics, study design, long COVID related data, cognitive outcomes and neuropsychological tests will be gathered. Descriptive analyses, quantification with heat maps and narrative synthesis will be conducted for reporting of results.

This scoping review has been registered with the Open Science Framework (https://doi.org/10.17605/OSF.IO/JHFX6).

Ethics and Dissemination: Ethical approval is not required, as no participant is involved in the study. The findings will be disseminated as a publication in a scientific journal and within the professional network.

Strengths and Limitations

- This study aims to illuminate the prolonged cognitive sequelae of COVID-19, an area currently characterized by limited understanding.
- A comprehensive search strategy was established with field experts and a research librarian, adding to the robustness of the review.
- A limitation is the heterogeneity in the field, with varying definition of the syndrome, symptoms associated with it and non-uniform way of assessing cognitive functioning in this context.
- No analysis of quality will be performed, in accordance with scoping review conventions.

Keywords: Long COVID, Post-Acute COVID-19 Syndrome, Cognition, Neuropsychology, Neuroepidemiology, Psychometrics

For peer review only - http://bmjopen.bmj.com/site/about/guidelines.xhtml

Background

According to the World Health Organization (WHO), 10–20% of individuals who contract SARS-CoV-2 infection may go on to experience chronic symptoms (1), that might persist from weeks and up to years (2). The overarching label for persistent symptoms is 'long COVID' (2,3); alternatively, 'postacute sequelae of SARS-CoV-2 infection' (PASC) (4), 'Post COVID-19 Syndrome (PCS) (5,6) and 'Post COVID-19 Condition' (PCC) (7) have been proposed (2,3). Debates exploring consensus for a unified syndrome definition, labels and symptoms and their temporal evolution are currently ongoing. Establishing a unified model is challenging, particularly due to the varying severity, duration and overall heterogeneity of symptoms, the novelty of the field and a lack of unified assessment approaches used in studies (2,8), consequently also affecting the availability of healthcare management (3).

Attempts have been made to map out the characteristics of the post COVID-19 syndrome (9). Three core categories seem to dominate the clinical picture, namely cognitive, psychiatric and systemic issues (9). Consensus seems to exist for frequent symptoms of the systemic core, which includes fatigue, headaches, anosmia/dysgeusia, myalgia, and respiratory difficulties, as well as the psychiatric core, mainly involving anxiety and depression (9,10). Memory, executive functioning and attentional deficits have been reported to be at the core of cognitive complaints, as well as cognitive slowing, commonly termed "brain fog" (9).

By dividing the condition into temporal stages, a number of models have been put forth during the last three years to explain and categorize the status of chronic symptoms (2,4). The majority of reviews and studies up to now focus on the early and acute phases following infection (6–10). Conversely, there has been a less comprehensive examination, especially regarding the neurocognitive symptom core, for long COVID symptoms beyond these immediate stages (8,9,11).

It is thus important to understand the current extent of knowledge in the field by identifying which symptoms are most commonly present in patients with persistent post COVID-19 syndrome, often referred to as "long haulers." Because long COVID has recently been found to be related with gray matter structural changes (11) and permanent alteration of brain connectivity, even eleven months after infection (12), it is essential to map the exact cognitive decline associated with the persistent manifestation (13). Attention, executive and memory components seem to lie at the core of the difficulties (9), however there is currently no single, cohesive concept about their persistency, which exact deficits are present to what extent and with regards to which initial severity, or on how to measure long COVID associated cognition (8,9). It is necessary to provide clarification regarding the heterogeneous use of assessment techniques, confounding caused by coexisting conditions that were not tested for, and a lack of comparison to earlier tests or controls (9). This approach will help with the optimization of existing medical diagnostic techniques, subsequent treatment plans and rehabilitative strategies and add to our understanding of the exact components of long COVID associated cognitive impairment.

For peer review only - http://bmjopen.bmj.com/site/about/guidelines.xhtml

Objectives:

The aim of this scoping review is to map out the persistent cognitive sequelae associated with long COVID symptomatology in adults, six months or more after infection.

- Characterize patterns of long COVID cognitive sequelae over time, specifically chronic symptoms present after at least six months or beyond.
- Investigate whether the initial disease severity (asymptomatic, mild, moderate, severe) influences the cognitive outcomes.
- Report on neuropsychological tests for cognitive assessment that were used to assess the cognitive changes, to understand the conceptual boundaries of current findings.
- Elucidate current gaps in the literature.

Methods and analysis:

Protocol and Registration

The protocol is registered under the Open Science Framework with OSF (https://doi.org/10.17605/OSF.IO/JHFX6).

Design

To guide the overall conception and reasoning, the Arksey and O'Malley methodological framework (14) will be applied to the design and decision making process of the systematic search. Additionally, the methodological concept proposed by the National Institute for Health Research (NIHR) Service Delivery and Organisation Research and Development Programme (15) will be used, with a main focus on 1) clarifying conceptual working definitions and to determine current boundaries of the field and concept, 2) identifying existing knowledge and present gaps, and 3) mapping existing evidence (16). Reporting the work will be aligned with the PRISMA Guidelines for Systematic Reviews and the Preferred Reporting Items for Systematic reviews and Meta-Analyses extension for scoping reviews (PRISMA-ScR) (17) and will follow the rationale of the Preferred Reporting Items for Systematic Reviews and Meta-Analyses (PRISMA) 2020 statement update (18), in the context of a scoping study.

To the best of our knowledge, after a preliminary search in November 2023 of PubMed, Google Scholar, OSF, PROSPERO, and the Cochrane Database of Systematic Reviews, no previous or upcoming reviews or protocols with a comparable design have been identified.

Patient and public involvement

For peer review only - http://bmjopen.bmj.com/site/about/guidelines.xhtml

No patients involved.

Eligibility Criteria

As shown in Table 1, the PICO Framework (19) served as a framework for determining the eligibility requirements.

Table 1: Eligibility Criteria based on the PICO Framework.

Element	Inclusion criteria	Exclusion criteria
**P**articipants	Human participants<br><br>Articles involving adults ≥18 years old<br><br>Contains reliable information on previous COVID-19 infection<br><br>Chronic, recurrent, or onset of cognitive symptomatology ≥6 months after infection	Animal studies<br><br>Articles focusing on patients <18 years old
**I**ntervention or exposure of interest	No intervention reviewed; exposure is 1) SARS-CoV-2 initial infection, 2) cognitive testing after 6 months<br><br>Chronic, recurrent, or onset of cognitive symptomatology ≥6 months after infection<br><br>Preferred inclusion of studies reporting exclusion of people with pre-infection cognitive symptoms or disorders<br><br>Included studies report on tools used for identification of cognitive symptoms<br><br>At least 1 objective, validated and quantifiable cognitive assessment tool used	Articles only reporting outcomes for symptom duration <6 months<br><br>Articles which do not include cognitive symptoms
**C**omparator	The studies should include cognition outcomes long time after COVID-19<br><br>Different cognitive symptoms of interest<br><br>Different types of initial disease severity (which could have required different types of medical	No information available about long COVID after 6 months or beyond<br><br>No initial infection to SARS-CoV-2

For peer review only - http://bmjopen.bmj.com/site/about/guidelines.xhtml

	care, e.g. hospitalisation)<br><br>Different assessment modalities for cognitive performance	
**O**utcome	Any neurocognitive outcome related to long COVID characteristics<br><br>“Long COVID” is not restricted to a specific definition or symptomatology, but exploration thereof remains open and will be explored<br><br>Alternative labels will be accepted<br><br>Any objective cognitive assessment modality will be included	Self-report of cognitive ability without objective measurement tool
Study design	Any type of study that assists in scoping the field, independent of the type, will be included (longitudinal-, transversal-, with a cohort or a case-control-design, review, meta analysis)	Studies with n<10 (e.g. case reports), qualitative studies
Article Type	Peer-reviewed articles<br><br>Available in full text	Grey literature, protocols, editorials, dissertations, book chapters, conference abstracts
Language	English manuscripts	
Study Period	From 2020	
Geography	No restriction	

For peer review only

Information resources

Articles will be extracted from the following databases:

- PubMed
- EMBASE

Search strategy

The search strategy was constructed together with a scientific librarian, to conduct a wide-ranging search within the selected databases, aiming at identification of an unbiased body of records. As outlined in Table 2, search terms from three themes (‘long COVID’, ‘cognition’ and ‘assessment’)

For peer review only - http://bmjopen.bmj.com/site/about/guidelines.xhtml

will be conceptually arranged to be placed in comprehensive search strings for selection of relevant articles. Within a theme, search terms are arranged in conceptual clusters, which will be combined by syntax techniques such as Boolean operators (‘OR’, ‘AND’), search tags (MeSH, title only) and filters (‘most recent’, ‘humans’, ‘english language’). Additionally, word truncation (*) will be used to capture alternate word-endings.

The precise search strings generated from this technique were applied to each database and will be documented and reported in terms of the PRISMA-ScR Framework (17) in the final review article.

Primary Search

A primary search conducted on December 21, 2023, resulted in the identification of 1409 articles. After an automated duplicate screening, 997 remained and were added to EndNote 21 to manage references and produce a bibliography for future screening.

Screening and Study Selection

Following the identification of articles matching the search strategy and duplicate removal, references in their totality will be documented and managed with the review software Rayyan.ai (20), articles will be systematically screened for eligibility in a stepwise manner by the reviewers. The number or studies after each step and exclusion decisions will be recorded, as illsutrated in Graph 1.

1) Eligibility by title and abstract: Two independent reviewers will be screening for titles and abstracts of the article, to identify eligibility for inclusion. The screening decisions will be compared between the two reviewers. A third independent reviewer will be consulted for any remaining discrepancies.
2) Full text screening: The reviewer pair will assess the article selection for their full texts. Articles will be excluded if 1) data of interest is not reported, 2) patient population not eligible, 3) non-preferred study design, 4) non-preferred article type, or any other non-alignment with the eligibility criteria. Discrepancies will be solved as described in the previous section.

For peer review only - http://bmjopen.bmj.com/site/about/guidelines.xhtml

Graph 1: Flow Diagram of the Study Selection Methodology in Concordance with the Reporting Items for Systematic Reviews and Meta-Analyses (PRISMA 2020) (18).

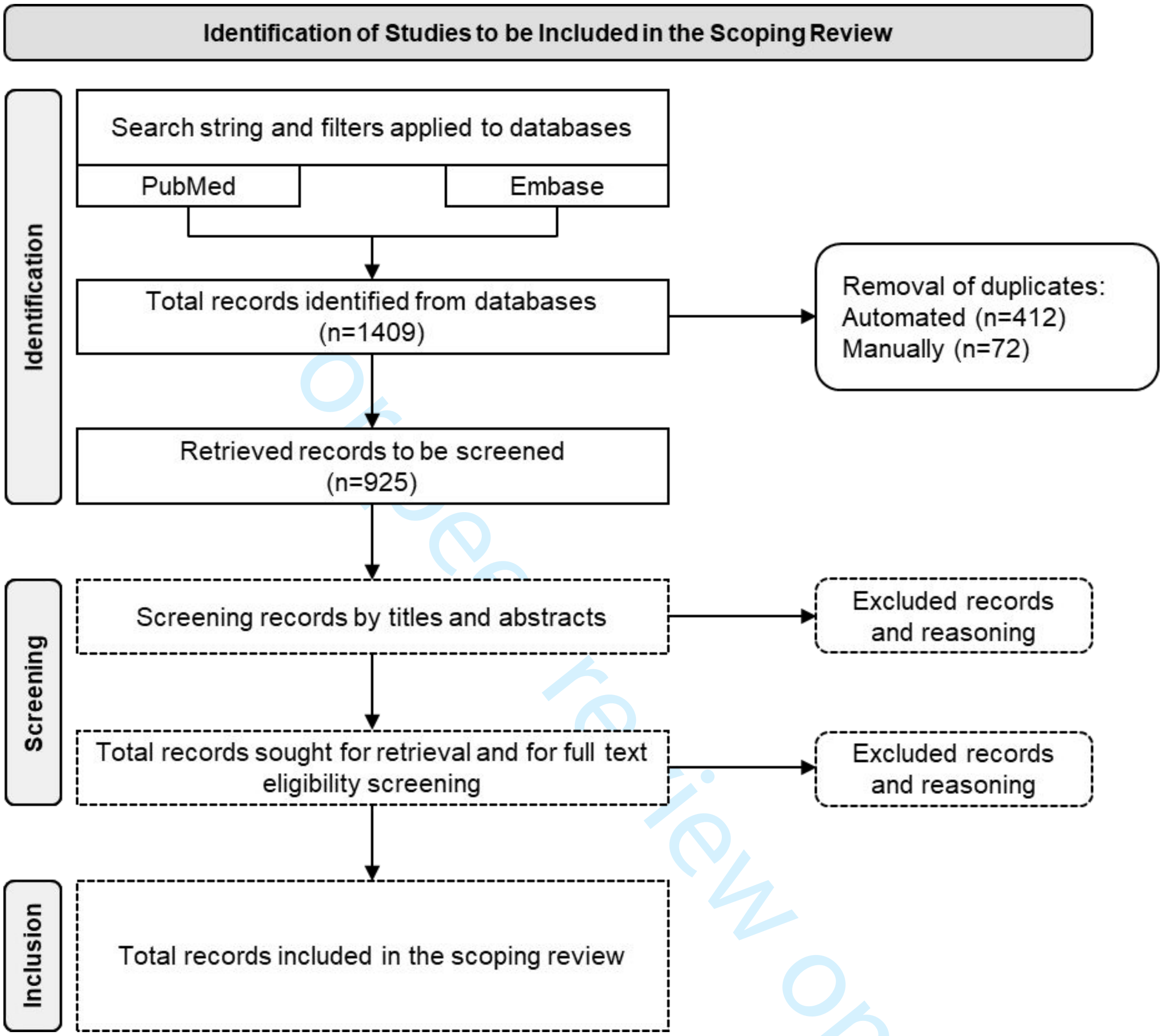

For peer review only - http://bmjopen.bmj.com/site/about/guidelines.xhtml

Table 2: Conceptual Search Strategy and Search Terms.

Theme	Key Concept		Search Terms/ Strings
**Long COVID**	**COVID**	**#1**	"COVID*" OR "SARS-CoV-2"
	**Infection**	**#2**	"COVID-19" OR "SARS-CoV-2"
	**Persistence**	**#3**	"long" OR "persistent" OR "chronic" OR "post-acute" OR "sequela*"
	**Long COVID**	**#4**	"Post-Acute COVID-19 Syndrome"
**Cognition**	**Memory**	**#5**	"memor*"
	**Attention**	**#6**	"attention"
	**Executive Functioning**	**#7**	"executive function*"
	**Vision**	**#8**	"visual" OR "visuo*"
	**Language**	**#9**	"language" OR "verbal" OR "fluency" OR "speech"
	**Cognition**	**#10**	"cogniti*"
	**Mental State**	**#11**	"brain fog" OR "confusion" OR "orientation" OR "disorientation"
**Assessment**	**Paradigms**	**#12**	"neuropsycholog*" OR "neurocogniti*"
	**Testbatteries**	**#13**	"MMSE" OR "MoCA" OR "CERAD" OR "TMT" OR "SDMT" OR "symbol digit modalities test" OR "Stroop" OR "N-BACK" OR "GO-NO*" OR "STMB*

For peer review only - http://bmjopen.bmj.com/site/about/guidelines.xhtml

Data management (included and excluded papers)

To maximize and document the objectivity of the screening process, the decisions and justifications for inclusion or exclusion will be documented in a digitalized spreadsheet table. Reference management, bibliography generation and write-up will be assisted by usage of EndNote 21, the screening will be conducted with Rayyan.ai (20) and a digital spread sheet will be used for charting the data.

Data collection process and extraction (Charting)

Rayyan.ai (20) and a digital spreadsheet will be utilized to extract and chart the data among the reviewer team members. All included papers will be reviewed by two reviewers, each independently charting data for the following variables:

- Author(s)
- Year of publication
- Title
- DOI
- Study objective
- Study design (e.g. cohort, multi-center studies, cross-sectional, longitudinal etc.)
- Country of study
- Setting context (e.g. acute, primary health care, long term care, home or lab setting, etc.)
- Sample size
- Patient demographics (sex/gender (proportion), age, education (if present),
- Diagnostic group (if applicable; asymptomatic, mild, moderate, severe), co-morbidity (if present), other characteristics related to long COVID etc.)
- Symptom outcomes and time points or ranges (6, 12, 18, 24, or more, months)
- Outcome definition (including diagnostic criteria if available)
- Cognitive assessment methodology, measurement tools used, classification outcomes (with raw scores if included), rationale for including tests if stated

For peer review only - http://bmjopen.bmj.com/site/about/guidelines.xhtml

- Statistical approach used
- The authors' primary conclusions and, if available, explanations of the factors taken into consideration during the decision-making process

The relevant items were adapted from the Cochrane Handbook for Systematic Reviews of Interventions (21).

Data analysis

To facilitate data synthesis, all the studies in review will be synthesized by being charted by the reviewers, to document the data of interest for reviewed studies and to visualize in the final report. The charted data will then be used to compute descriptive statistics and heat maps. This approach will support a quantifiable characterization of the concepts, which aids at giving insights into the field and objectives, given the sufficiency of collected data. Outcomes will be visualized in graphs and figures. The findings and figures will finally be disseminated in a narrative report.

Quality Assessment

In accordance with the guidelines developed by the Joanna Briggs Institute (JBI) for scoping reviews (22), no assessment of quality for included papers will be performed.

For peer review only

For peer review only - http://bmjopen.bmj.com/site/about/guidelines.xhtml

Contributors: Conceiving the idea of the scoping review and protocol – SM. Design of the protocol – SM, MP. Search strategy – SM, CD, MP. Draft of the manuscript – SM. Review and final approval of the manuscript – MP.

Funding: This work is supported by the Luxembourg National Research Fund (FNR), Project n°20210613.

Ethics: Given the use of secondary data, no ethical approval will be required, as no participants will be involved in this research.

Peer review: External peer review

Competing interests: None declared

Implications: This scoping review aims to map out the present field of long COVID cognition by adding novel insights into the characteristics of persistent cognitive decline symptoms for long COVID and investigating associated symptomatology and current testing conventions. By exploring the current scope of the field, valuable new insights can be added to better understand the nuanced dimensions of long COVID related cognitive decline and tailor healthcare interventions and rehabilitation programs accordingly.

Open access: This research was funded in whole by the Luxembourg National Research Fund (FNR), grant reference 16758430. For the purpose of open access, and in fulfilment of the obligations arising from the grant agreement, the author has applied a Creative Commons Attribution-ShareAlike 4.0 International (CC BY-SA 4.0) license to any Author Accepted Manuscript version arising from this submission. See: https://creativecommons.org/licenses/by-sa/4.0/deed.en

For peer review only

References:

1. Post COVID-19 condition (Long COVID) [Internet]. [cited 2023 Dec 27]. Available from: https://www.who.int/europe/news-room/fact-sheets/item/post-covid-19-condition

2. Munblit D, O'Hara ME, Akrami A, Perego E, Olliaro P, Needham DM. Long COVID: aiming for a consensus. Lancet Respir Med. 2022 Jul;10(7):632–4.

3. Fernández-de-Las-Peñas C. Long COVID: current definition. Infection. 2022 Feb;50(1):285–6.

4. Thaweethai T, Jolley SE, Karlson EW, Levitan EB, Levy B, McComsey GA, et al. Development of a Definition of Postacute Sequelae of SARS-CoV-2 Infection. JAMA. 2023 Jun 13;329(22):1934–46.

5. Venkatesan P. NICE guideline on long COVID. Lancet Respir Med. 2021 Feb;9(2):129.

6. Shah W, Heightman M, O'Brien S. UK guidelines for managing long-term effects of COVID-19. Lancet. 2021 May 8;397(10286):1706.

7. Soriano JB, Murthy S, Marshall JC, Relan P, Diaz JV, WHO Clinical Case Definition Working Group on Post-COVID-19 Condition. A clinical case definition of post-COVID-19 condition by a Delphi consensus. Lancet Infect Dis. 2022 Apr;22(4):e102–7.

8. Sobrino-Relaño S, Balboa-Bandeira Y, Peña J, Ibarretxe-Bilbao N, Zubiaurre-Elorza L, Ojeda N. Neuropsychological deficits in patients with persistent COVID-19 symptoms: a systematic review and meta-analysis. Sci Rep. 2023 Jun 26;13(1):10309.

9. Nicotra A, Masserini F, Calcaterra F, Di Vito C, Doneddu PE, Pomati S, et al. What do we mean by long COVID? A scoping review of the cognitive sequelae of SARS-CoV-2 infection. Eur J Neurol. 2023 Dec;30(12):3968–78.

10. Hayes LD, Ingram J, Sculthorpe NF. More Than 100 Persistent Symptoms of SARS-CoV-2 (Long COVID): A Scoping Review. Front Med. 2021 Nov 1;8:750378.

11. Douaud G, Lee S, Alfaro-Almagro F, Arthofer C, Wang C, McCarthy P, et al. SARS-CoV-2 is associated with changes in brain structure in UK Biobank. Nature. 2022 Apr;604(7907):697–707.

12. Díez-Cirarda M, Yus M, Gómez-Ruiz N, Polidura C, Gil-Martínez L, Delgado-Alonso C, et al. Multimodal neuroimaging in post-COVID syndrome and correlation with cognition. Brain. 2023 May 2;146(5):2142–52.

13. Ferrucci R, Dini M, Rosci C, Capozza A, Groppo E, Reitano MR, et al. One-year cognitive follow-up of COVID-19 hospitalized patients. Eur J Neurol. 2022 Jul;29(7):2006–14.

14. Arksey H, O'Malley L. Scoping studies: towards a methodological framework. Int J Soc Res Methodol. 2005 Feb 1;8(1):19–32.

For peer review only - http://bmjopen.bmj.com/site/about/guidelines.xhtml

15. Anderson S, Allen P, Peckham S, Goodwin N. Asking the right questions: scoping studies in the commissioning of research on the organisation and delivery of health services. Health Res Policy Syst. 2008 Jul 9;6:7.

16. Levac D, Colquhoun H, O'Brien KK. Scoping studies: advancing the methodology. Implement Sci. 2010 Sep 20;5:69.

17. Tricco AC, Lillie E, Zarin W, O'Brien KK, Colquhoun H, Levac D, et al. PRISMA Extension for Scoping Reviews (PRISMA-ScR): Checklist and Explanation. Ann Intern Med. 2018 Oct 2;169(7):467–73.

18. Page MJ, McKenzie JE, Bossuyt PM, Boutron I, Hoffmann TC, Mulrow CD, et al. The PRISMA 2020 statement: an updated guideline for reporting systematic reviews. Rev Esp Cardiol . 2021 Sep;74(9):790–9.

19. Schardt C, Adams MB, Owens T, Keitz S, Fontelo P. Utilization of the PICO framework to improve searching PubMed for clinical questions. BMC Med Inform Decis Mak. 2007 Jun 15;7:16.

20. Ouzzani M, Hammady H, Fedorowicz Z, Elmagarmid A. Rayyan—a web and mobile app for systematic reviews. Syst Rev. 2016 Dec 5;5(1):210.

21. Cochrane Collaboration. Cochrane Handbook for Systematic Reviews of Interventions. Cochrane Collaboration; 2021.

22. Peters MDJ, Godfrey CM, Khalil H, McInerney P, Parker D, Soares CB. Guidance for conducting systematic scoping reviews. Int J Evid Based Healthc. 2015 Sep;13(3):141–6.

For peer review only

For peer review only - http://bmjopen.bmj.com/site/about/guidelines.xhtml

Graph 1: Flow Chart of the Screening Methodology in Concordance with the Reporting Items for Systematic Reviews and Meta-Analyses (PRISMA).

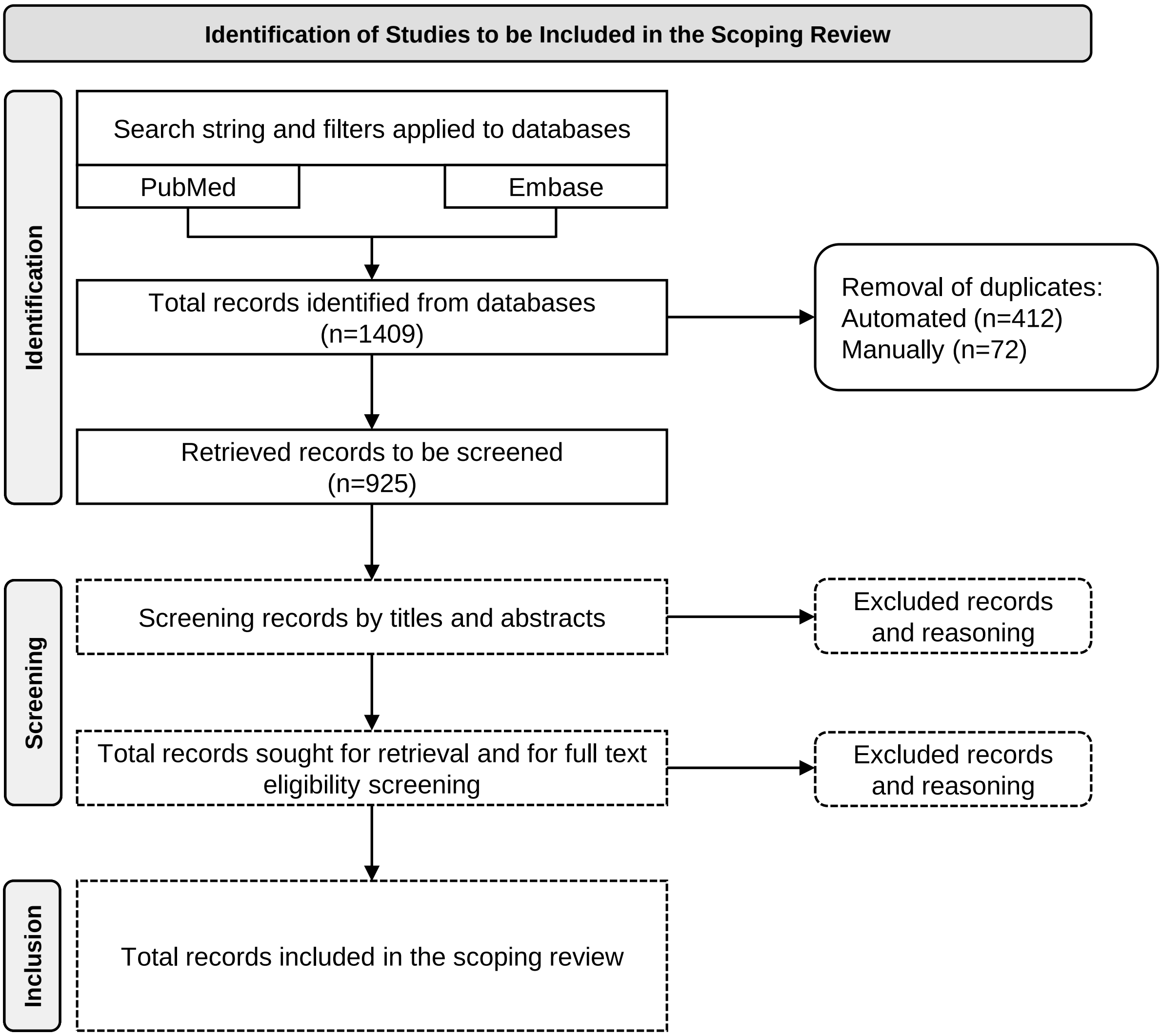

Table 1: Eligibility Criteria based on the PICO Framework.

Element	Inclusion criteria	Exclusion criteria
**P**articipants	Human participants<br><br>Articles involving adults ≥18 years old<br><br>Contains reliable information on previous COVID-19 infection<br><br>Chronic, recurrent, or onset of cognitive symptomatology ≥6 months after infection	Animal studies<br><br>Articles focusing on patients <18 years old
**I**ntervention or exposure of interest	No intervention reviewed; exposure is 1) SARS-CoV-2 initial infection, 2) cognitive testing after 6 months<br><br>Chronic, recurrent, or onset of cognitive symptomatology ≥6 months after infection<br><br>Preferred inclusion of studies reporting exclusion of people with pre-infection cognitive symptoms or disorders<br><br>Included studies report on tools used for identification of cognitive symptoms<br><br>At least 1 objective, validated and quantifiable cognitive assessment tool used	Articles only reporting outcomes for symptom duration <6 months<br><br>Articles which do not include cognitive symptoms
**C**omparator	The studies should include cognition outcomes long time after COVID-19<br><br>Different cognitive symptoms of interest<br><br>Different types of initial disease severity (which could have required different types of medical care, e.g. hospitalisation)<br><br>Different assessment modalities for cognitive performance	No information available about long COVID after 6 months or beyond<br><br>No initial infection to SARS-CoV-2
**O**utcome	Any neurocognitive outcome related to long COVID characteristics<br><br>“Long COVID” is not restricted to a specific definition or symptomatology, but exploration thereof remains open and will be explored<br><br>Alternative labels will be accepted<br><br>Any objective cognitive assessment modality will be included	Self-report of cognitive ability without objective measurement tool
Study design	Any type of study that assists in scoping the field, independent of the type, will be included (longitudinal-, transversal-, with a cohort or a case-control-design, review, meta analysis)	Studies with n<10 (e.g. case reports), qualitative studies
Article Type	Peer-reviewed articles<br><br>Available in full text	Grey literature, protocols, editorials, dissertations, book chapters, conference abstracts
Language	English manuscripts	
Study Period	From 2020	
Geography	No restriction	

For peer review only - http://bmjopen.bmj.com/site/about/guidelines.xhtml

Table 2: Conceptual Search Strategy and Search Terms.

Theme	Key Concept		Search Terms/ Strings
**Long COVID**	**COVID**	**#1**	"COVID*" OR "SARS-CoV-2"
	**Infection**	**#2**	"COVID-19" OR "SARS-CoV-2"
	**Persistence**	**#3**	"long" OR "persistent" OR "chronic" OR "post-acute" OR "sequela*"
	**Long COVID**	**#4**	"Post-Acute COVID-19 Syndrome"
**Cognition**	**Memory**	**#5**	"memor*"
	**Attention**	**#6**	"attention"
	**Executive Functioning**	**#7**	"executive function*"
	**Vision**	**#8**	"visual" OR "visuo*"
	**Language**	**#9**	"language" OR "verbal" OR "fluency" OR "speech"
	**Cognition**	**#10**	"cogniti*"
	**Mental State**	**#11**	"brain fog" OR "confusion" OR "orientation" OR "disorientation"
**Assessment**	**Paradigms**	**#12**	"neuropsycholog*" OR "neurocogniti*"
	**Testbatteries**	**#13**	"MMSE" OR "MoCA" OR "CERAD" OR "TMT" OR "SDMT" OR "symbol digit modalities test" OR "Stroop" OR "N-BACK" OR "GO-NO*" OR "STMB*

Reporting checklist for protocol of a systematic review and meta analysis.

Based on the PRISMA-P guidelines.

Moher D, Shamseer L, Clarke M, Ghersi D, Liberati A, Petticrew M, Shekelle P, Stewart LA. Preferred Reporting Items for Systematic Review and Meta-Analysis Protocols (PRISMA-P) 2015 statement. Syst Rev. 2015;4(1):1.

		Reporting Item	Page Number
**Title**			
Identification	#1a	Identify the report as a protocol of a systematic review	1
Update	#1b	If the protocol is for an update of a previous systematic review, identify as such	n/a
**Registration**			
	#2	If registered, provide the name of the registry (such as PROSPERO) and registration number	2
**Authors**			
Contact	#3a	Provide name, institutional affiliation, e-mail address of all protocol authors; provide physical mailing address of corresponding author	1
Contribution	#3b	Describe contributions of protocol authors and identify the guarantor of the review	12
**Amendments**			
	#4	If the protocol represents an amendment of a previously completed or published protocol, identify as such and list changes; otherwise, state plan for documenting important protocol amendments	n/a
**Support**			
Sources	#5a	Indicate sources of financial or other support for the review	12

For peer review only

Sponsor	#5b	Provide name for the review funder and / or sponsor	12
Role of sponsor or funder	#5c	Describe roles of funder(s), sponsor(s), and / or institution(s), if any, in developing the protocol	12
**Introduction**			
Rationale	#6	Describe the rationale for the review in the context of what is already known	3
Objectives	#7	Provide an explicit statement of the question(s) the review will address with reference to participants, interventions, comparators, and outcomes (PICO)	4
**Methods**			
Eligibility criteria	#8	Specify the study characteristics (such as PICO, study design, setting, time frame) and report characteristics (such as years considered, language, publication status) to be used as criteria for eligibility for the review	4-6
Information sources	#9	Describe all intended information sources (such as electronic databases, contact with study authors, trial registers or other grey literature sources) with planned dates of coverage	6
Search strategy	#10	Present draft of search strategy to be used for at least one electronic database, including planned limits, such that it could be repeated	6-9
Study records - data management	#11a	Describe the mechanism(s) that will be used to manage records and data throughout the review	10
Study records - selection process	#11b	State the process that will be used for selecting studies (such as two independent reviewers) through each phase of the review (that is, screening, eligibility and inclusion in meta-analysis)	7
Study records - data collection process	#11c	Describe planned method of extracting data from reports (such as piloting forms, done independently, in duplicate), any processes for obtaining and confirming data from investigators	10-11
Data items	#12	List and define all variables for which data will be sought (such as PICO items, funding sources), any pre-planned data assumptions and simplifications	10-11

For peer review only

Outcomes and prioritization	#13	List and define all outcomes for which data will be sought, including prioritization of main and additional outcomes, with rationale	10-11
Risk of bias in individual studies	#14	Describe anticipated methods for assessing risk of bias of individual studies, including whether this will be done at the outcome or study level, or both; state how this information will be used in data synthesis	11
Data synthesis	#15a	Describe criteria under which study data will be quantitatively synthesised	11
Data synthesis	#15b	If data are appropriate for quantitative synthesis, describe planned summary measures, methods of handling data and methods of combining data from studies, including any planned exploration of consistency (such as I2, Kendall's τ)	11
Data synthesis	#15c	Describe any proposed additional analyses (such as sensitivity or subgroup analyses, meta-regression)	11
Data synthesis	#15d	If quantitative synthesis is not appropriate, describe the type of summary planned	11
Meta-bias(es)	#16	Specify any planned assessment of meta-bias(es) (such as publication bias across studies, selective reporting within studies)	10-11
Confidence in cumulative evidence	#17	Describe how the strength of the body of evidence will be assessed (such as GRADE)	11

For peer review only

Long COVID Cognitive Sequelae 6 Months Post-Infection and Beyond: A Scoping Review Protocol

Journal:	*BMJ Open*
Manuscript ID	bmjopen-2024-084798.R1
Article Type:	Protocol
Date Submitted by the Author:	11-Apr-2024
Complete List of Authors:	Monteiro, Sara; Luxembourg Institute of Health, Department of Precision Health<br>Dessenne, Coralie; Luxembourg Institute of Health, Science Office<br>Perquin, Magali ; Luxembourg Institute of Health, Department of Precision Health
**Primary Subject Heading**:	Public health
Secondary Subject Heading:	Mental health, Neurology, Infectious diseases, Research methods
Keywords:	Post-Acute COVID-19 Syndrome, Psychometrics, Chronic Disease, COVID-19, EPIDEMIOLOGY, MENTAL HEALTH

SCHOLARONE™
Manuscripts

Long COVID Cognitive Sequelae 6 Months Post Infection and Beyond:

A Scoping Review Protocol

Sara Monteiro[1,*], Coralie Dessenne[2], Magali Perquin[1].

[1] Department of Precision Health, Luxembourg Institute of Health, 1A-B, rue Thomas Edison, 1445 Strassen, Luxembourg

[2] Science Office, Luxembourg Institute of Health, 1A-B, rue Thomas Edison, 1445 Strassen, Luxembourg

***Corresponding Author:**

Sara Monteiro: sara.monteiro@lih.lu, ORCID: 0000-0001-9145-0063

Author Note:

Coralie Dessenne: coralie.dessenne@lih.lu, ORCID: 0000-0001-7723-0304

Magali Perquin: magali.perquin@lih.lu, ORCID: 0000-0002-0386-0212

Declaration: No conflict of interest to declare.

For peer review only

Abstract

Introduction: The novel and expanding field of long COVID research has undergone diverse methodological approaches in recent years. This protocol lays out the methodological approach, which aims at identifying nuances in current research. It underscores the necessity for a more precise understanding of prolonged cognitive sequelae and their relation to initial disease severity. The findings will add valuable insights for the development of targeted rehabilitation, healthcare interventions and thereby aid patients, clinicians, policymakers and researchers. Our upcoming research is hereby introduced.

Methods and Analysis: To map current research in the field, a scoping review will be conducted and documented in accordance with the Preferred Reporting Items for Systematic Reviews and Meta-Analyses - Scoping Review Extension standards. A systematic search of scientific databases (PubMed, EMBASE), presented 1409 eligible results, published up to 21 December 2023. After removal of duplicates, 925 articles were extracted for screening. Two independent reviewers will screen for titles, abstracts and full texts, to extract data, which will then be organized using charting software. Data for various variables, i.e. journal info, studied population demographics, study design, long COVID related data, cognitive outcomes and neuropsychological tests will be gathered. Descriptive analyses, Evidence Gap Maps (EGM), heat map quantifications and narrative synthesis will be conducted for reporting of results.

This scoping review has been registered with the Open Science Framework (https://doi.org/10.17605/OSF.IO/JHFX6).

Ethics and Dissemination: Ethical approval is not required, as the study does not involve human participants. The findings will be disseminated through a publication in a scientific journal and within the professional network.

Strengths and Limitations

- Cognitive symptoms will be characterized in detail, highlighting domains and particular sub-skills for in depth understanding of long COVID pathology.
- The multifaceted approach used (in-depth psychometrics, acute, and long-term COVID-19 symptomatology, as well as comorbid factors), enables a nuanced analysis and increases accuracy.
- A multidisciplinary team covering Information Science, Neuropsychology and Epidemiology add to the depth of interpretability and robustness of the review, by establishing a comprehensive search strategy and evaluation.
- Evidence maps will be used to address present heterogeneity in the field and thereby allowing for improved scientific understanding and enhanced clinical interventions.

Keywords: Long COVID, Post-Acute COVID-19 Syndrome, Cognition, Neuropsychology, Neuroepidemiology, Psychometrics

For peer review only - http://bmjopen.bmj.com/site/about/guidelines.xhtml

Background

According to the World Health Organization (WHO), 10–20% of individuals who contract SARS-CoV-2 infection may go on to experience chronic symptoms (1), that might persist from weeks and up to years (2). The overarching label for persistent symptoms is 'long COVID' (2,3); alternatively, 'post-acute sequelae of SARS-CoV-2 infection' (PASC) (4), 'Post COVID-19 Syndrome (PCS) (5,6) and 'Post COVID-19 Condition' (PCC) (7) have been proposed (2,3). Debates exploring consensus for a unified syndrome definition, labels and symptoms and their temporal evolution are currently ongoing. Establishing a unified model is challenging, particularly due to the varying severity, duration and overall heterogeneity of symptoms, the novelty of the field and a lack of unified assessment approaches used in studies (2,8), consequently also affecting the availability of healthcare management (3).

Attempts have been made to map out the characteristics of the post COVID-19 syndrome (9). Three core categories seem to dominate the clinical picture, namely cognitive, psychiatric and systemic issues (9). Consensus seems to exist for frequent symptoms of the systemic core, which includes fatigue, headaches, anosmia/ dysgeusia, myalgia, and respiratory difficulties, as well as the psychiatric core, mainly involving anxiety and depression (9,10). Memory, executive functioning and attentional deficits have been reported to be at the core of cognitive complaints, as well as cognitive slowing, commonly termed "brain fog" (9).

By dividing the condition into temporal stages, a number of models have been put forth during the last three years to explain and categorize the status of chronic symptoms (2,4). The majority of reviews and studies up to now focus on the early and acute phases following infection (6–10). Recent scoping reviews on COVID-19 reported cognitive symptoms and tests being used for screening up to six months maximum after the acute phase (9), as well as psychiatric conditions prior to and immediately after infection (12, 13). Conversely, there has been a less comprehensive examination, especially regarding the neurocognitive symptom core, for long COVID symptoms beyond these immediate stages (8, 9, 11). However, a comparison between immediate and long-term effects has been reviewed, albeit on specific populations, such as patients with substance use disorders (14). Thus, although existing, to our knowledge, no study has addressed the specific and recurrent cognitive symptoms beyond 6 months after infection.

It is thus important to improve understanding of the current gap in the field relating to cognitive long-term sequelae, by identifying which symptoms are most commonly present in patients often referred to as "long haulers." Because long COVID has recently been found to be related with gray matter structural changes (11) and permanent alteration of brain connectivity, even eleven months after infection (15), it is essential to map the exact cognitive decline associated with the persistent manifestation (16). Attention, executive and memory components seem to lie at the core of the difficulties (9), however there is currently no single, cohesive concept about their persistency, which exact deficits are present to what extent and with regards to which initial severity, or on how to measure long COVID associated cognition (8,9). Additionally, it is

For peer review only - http://bmjopen.bmj.com/site/about/guidelines.xhtml

necessary to provide clarification regarding the heterogeneous use of study designs, assessment techniques, confounding caused by coexisting conditions that were not tested for, and a lack of comparison to earlier tests or controls (9). This approach will help with the optimization of existing medical diagnostic techniques, subsequent treatment plans and rehabilitative strategies and add to our understanding of the exact components of long COVID associated cognitive impairment.

Objectives:

The aim of this scoping review is to map out the persistent cognitive sequelae associated with long COVID symptomatology in adults, six months or more after infection.

- Characterize patterns of long COVID cognitive sequelae over time, specifically chronic symptoms present after at least six months or beyond.
- Investigate whether the initial disease severity (asymptomatic, mild, moderate, severe) influences the cognitive outcomes.
- Report on neuropsychological tests for cognitive assessment that were used to assess the cognitive changes, to understand the conceptual boundaries of current findings.
- Elucidate current gaps in the literature.

Methods and analysis:

Protocol and Registration

The protocol is registered under the Open Science Framework with OSF (https://doi.org/10.17605/OSF.IO/JHFX6).

Design

To guide the overall conception and reasoning, the Arksey and O'Malley methodological framework (17) will be applied to the design and decision making process of the systematic search. Additionally, the methodological concept proposed by the National Institute for Health Research (NIHR) Service Delivery and Organisation Research and Development Programme (18) will be used, with a main focus on 1) clarifying conceptual working definitions and to determine current boundaries of the field and concept, 2) identifying existing knowledge and present gaps, and 3) mapping existing evidence (19). Reporting the work will be aligned with the PRISMA Guidelines for Systematic Reviews and the Preferred Reporting Items for Systematic reviews and Meta-Analyses extension for scoping reviews (PRISMA-ScR) (20) and will follow the rationale of the Preferred Reporting Items for Systematic Reviews and Meta-Analyses (PRISMA) 2020 statement update (21), in the context of a scoping study.

For peer review only - http://bmjopen.bmj.com/site/about/guidelines.xhtml

To the best of our knowledge, after a preliminary search in November 2023 of PubMed, Google Scholar, OSF, PROSPERO, and the Cochrane Database of Systematic Reviews, no previous or upcoming reviews or protocols with a comparable design have been identified.

Patient and public involvement

No patients involved.

Eligibility Criteria

As shown in Table 1, the PICO Framework (22) served as a framework for determining the eligibility requirements.

Table 1: Eligibility Criteria Based on the PICO Framework.

Element	Inclusion criteria	Exclusion criteria
**P**articipants	Human participants<br><br>Articles involving adults ≥18 years old<br><br>Contains reliable information on previous COVID-19 infection<br><br>Chronic, recurrent, or onset of cognitive symptomatology ≥6 months after infection	Animal studies<br><br>Articles focusing on patients <18 years old
**I**ntervention or exposure of interest	No intervention reviewed; exposure is 1) SARS-CoV-2 initial infection, 2) cognitive testing after 6 months<br><br>Chronic, recurrent, or onset of cognitive symptomatology ≥6 months after infection<br><br>Preferred inclusion of studies reporting exclusion of people with pre-infection cognitive symptoms or disorders<br><br>Included studies report on tools used for identification of cognitive symptoms<br><br>At least 1 objective, validated and quantifiable cognitive assessment tool used	Articles only reporting outcomes for symptom duration <6 months<br><br>Articles which do not include cognitive symptoms

For peer review only

For peer review only - http://bmjopen.bmj.com/site/about/guidelines.xhtml

**C**omparator	The studies should include cognition outcomes long time after COVID-19<br><br>Different cognitive symptoms of interest<br><br>Different types of initial disease severity (which could have required different types of medical care, e.g. hospitalisation)<br><br>Different assessment modalities for cognitive performance	No information available about long COVID after 6 months or beyond<br><br>No initial infection to SARS-CoV-2
**O**utcome	Any neurocognitive outcome related to long COVID characteristics<br><br>“Long COVID” is not restricted to a specific definition or symptomatology, but exploration thereof remains open and will be explored<br><br>Alternative labels will be accepted<br><br>Any objective cognitive assessment modality will be included	Self-report of cognitive ability without objective measurement tool
Study design	Any type of study that assists in scoping the field, independent of the type, will be included (preference for longitudinal-, transversal-, with a cohort or a case-control-design, review, meta-analysis)	Studies with n<10 (e.g. case reports), qualitative studies
Article Type	Peer-reviewed articles<br><br>Available in full text	Grey literature, protocols, editorials, dissertations, book chapters, conference abstracts
Language	English manuscripts	
Study Period	From 2020	
Geography	No restriction	

1 Information resources

2 Articles will be extracted from the following databases:

3 - PubMed

For peer review only - http://bmjopen.bmj.com/site/about/guidelines.xhtml

- EMBASE

Search strategy

The search strategy was constructed together with a scientific librarian, to conduct a wide-ranging search within the selected databases, aiming at identification of an unbiased body of records. As outlined in Table 2, search terms from three themes ('long COVID', 'cognition' and 'assessment') will be conceptually arranged to be placed in comprehensive search strings for selection of relevant articles. Within a theme, search terms are arranged in conceptual clusters, which will be combined by syntax techniques such as Boolean operators ('OR', 'AND'), search tags (MeSH, title only) and filters ('humans', 'English language'). Additionally, word truncation (*) will be used to capture alternate word-endings. For example, the search term "COVID*" will capture instances of the word "COVID," and also include other labels or terms containing "COVID" within them (e.g. "long-COVID", "post-COVID", "COVID-19" etc.). Similarly, the search term "Visuo*" will include "Visuo-spatial", "Visuospatial", "Visuo-constructive", etc.

The precise search strings generated from this technique were applied to each database and will be documented and reported in terms of the PRISMA-ScR Framework (20) in the final review article.

Primary Search

A primary search conducted on December 21, 2023, resulted in the identification of 1409 articles. After an automated duplicate screening, 997 remained and were added to EndNote 21 to manage references and produce a bibliography for future screening. 925 records remained for screening after manual duplicate deletion.

Screening and Study Selection

The review will be conducted in six stages: the problem is identified and the study design is being developed, the search strategy is established and implemented, literature screening, data collection, data analysis, write-up and dissemination of findings.

Following the identification of articles matching the search strategy and duplicate removal, references in their totality will be documented and managed with the review software Rayyan.ai (23). Articles will be systematically screened for eligibility in a stepwise manner by the reviewers. The number or studies after each step and exclusion decisions will be recorded, as illustrated in Graph 1.

1) Eligibility by title and abstract: Two independent reviewers will be screening for titles and abstracts of the article, to identify eligibility for inclusion. The screening decisions will be compared between the two reviewers. A third independent reviewer will be consulted for any remaining discrepancies.

For peer review only - http://bmjopen.bmj.com/site/about/guidelines.xhtml

2) Full text screening: The reviewer pair will assess the article selection for their full texts. Articles will be excluded if 1) data of interest is not reported, 2) patient population not eligible, 3) non-preferred study design, 4) non-preferred article type, or any other non-alignment with the eligibility criteria. Discrepancies will be solved as described in the

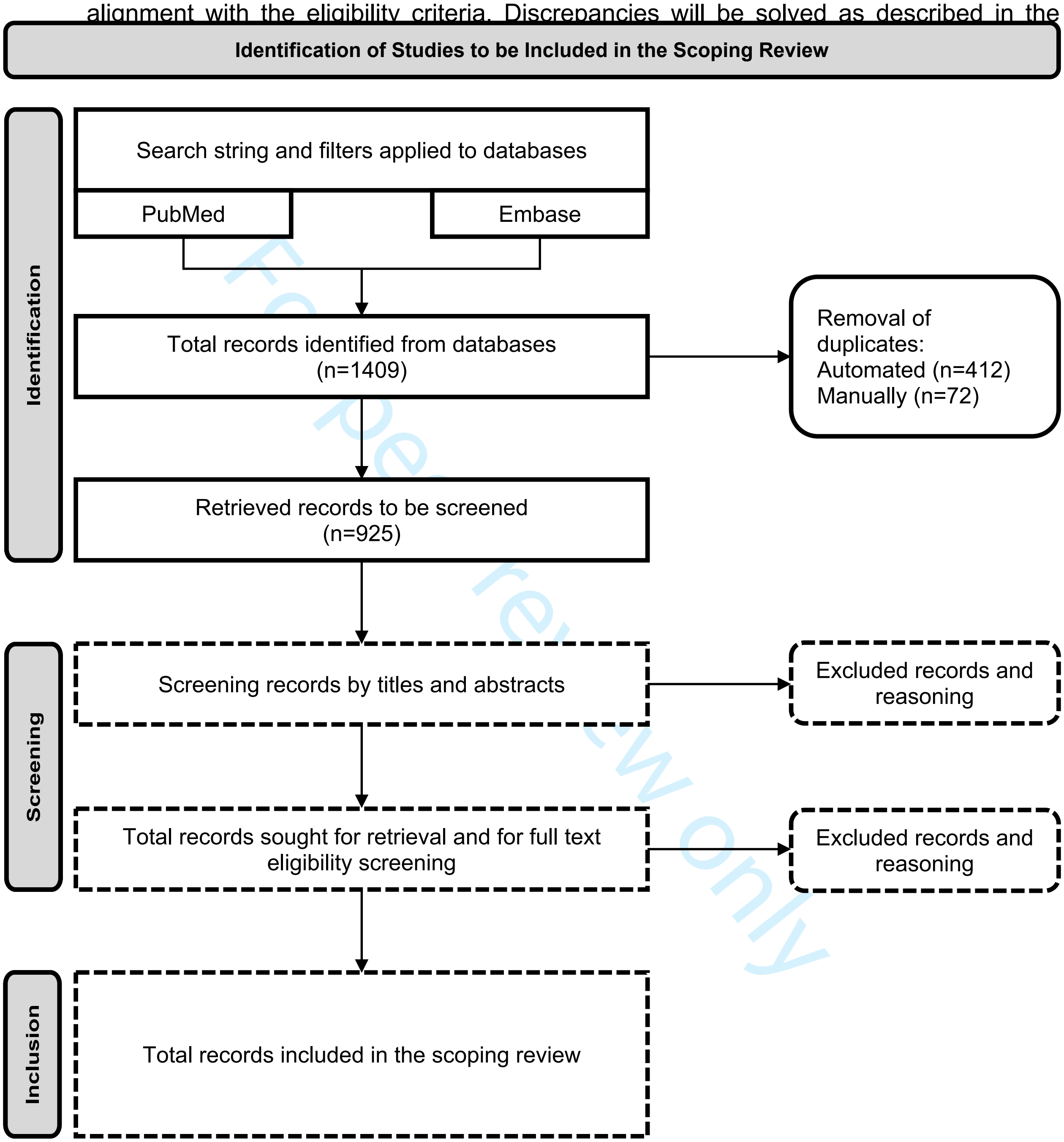

For peer review only - http://bmjopen.bmj.com/site/about/guidelines.xhtml

Figure 1: Flow Diagram of the Study Selection Methodology in Concordance with the Reporting Items for Systematic Reviews and Meta-Analyses (PRISMA 2020) (21).

Table 2: **Conceptual Search Strategy and Search Terms.**
The Theme column specifies the specific topics of focus. The Key Concept column outlines the different notions covered by each corresponding Theme. The Search Terms/Strings column provides:

- The terms in the article either in any field, either in a specific field such as the Title,
- The exact standardized terms to index articles in the databases - they are assigned to articles to categorize their content in a consistent and organized manner. These standardized terms are known as MeSH terms (Medical Subject Headings) in MEDLINE/PubMed and EMTREE terms in EMBASE.

**Theme**	**Key Concept**		**Search Terms/ Strings in PubMed**	**Search Terms/ Strings in EMBASE**
**Long COVID**	**COVID**	**#1**	"COVID*"[TI] OR "SARS-CoV-2"[TI]	'covid*':ti OR 'sars-cov-2':ti
	**Infection**	**#2**	"COVID-19"[Mesh] OR "SARS-CoV-2"[Mesh]	'coronavirus disease 2019'/exp
	**Persistence**	**#3**	"long" OR "persistent" OR "chronic" OR "post-acute" OR "sequela*"	'long' OR 'persistent' OR 'chronic' OR 'post-acute' OR 'sequela*'
	**Long COVID**	**#4**	"Post-Acute COVID-19 Syndrome"[Mesh]	'long covid'/exp
**Cognition**	**Memory**	**#5**	"memor*"[TI]	'memor*':ti
	**Attention**	**#6**	"attention"[TI]	'attention':ti
	**Executive Functioning**	**#7**	"executive function*"[TI]	'executive function*'
	**Vision**	**#8**	"visual" OR "visuo*"[TI]	'visual':ti OR 'visuo*':ti

For peer review only

For peer review only - http://bmjopen.bmj.com/site/about/guidelines.xhtml

	**Language**	**#9**	"language"[TI] OR "verbal"[TI] OR "fluency"[TI] OR "speech"[TI]	'language':ti OR 'verbal':ti OR 'fluency':ti OR 'speech':ti
	**Cognition**	**#10**	"cogniti*"[TI]	'cogniti*':ti
	**Mental State**	**#11**	"brain fog"[TI] OR "confusion"[TI] OR "orientation"[TI] OR "disorientation"[TI]	'brain fog':ti OR 'confusion':ti OR 'orientation':ti OR 'disorientation':ti
**Assessment**	**Paradigms**	**#12**	"neuropsycholog*"[TI] OR "neurocogniti*"[TI]	'neuropsycholog*':ti OR 'neurocogniti*':ti
	**Testbatteries**	**#13**	"MMSE" OR "MoCA" OR "CERAD" OR "TMT" OR "SDMT" OR "symbol digit modalities test" OR "Stroop" OR "N-BACK" OR "GO-NO*" OR "STMB*	'mmse' OR 'moca' OR 'cerad' OR 'tmt' OR 'sdmt' OR 'symbol digit modalities test' OR 'stroop' OR 'n-back' OR 'go-no*' OR 'stmb*'

The final strategy combined successively all these search step as follows:

(((#1 OR #2) AND #3) OR #4) AND (#5 OR #6 OR #7 OR #8 OR #9 OR #10 OR #11 OR #12 OR #13)

<u>Data management (included and excluded papers)</u>

To maximize and document the objectivity of the screening process, the decisions and justifications for inclusion or exclusion will be documented in a digitalized spreadsheet table. Reference management, bibliography generation and write-up will be assisted by usage of EndNote 21, the screening will be conducted with Rayyan.ai (23) and a digital spread sheet will be used for charting the data.

<u>Data collection process and extraction (Charting)</u>

Rayyan.ai (23) and a digital spreadsheet will be utilized to extract and chart the data among the reviewer team members. All included papers will be reviewed by two reviewers, each independently charting data for the following variables:

For peer review only - http://bmjopen.bmj.com/site/about/guidelines.xhtml

For peer review only

- Author(s)
- Year of publication
- Title
- DOI
- Study objective
- Study design (e.g. cohort, multi-center studies, cross-sectional, longitudinal etc.)
- Country of study
- Setting context (e.g. acute, primary health care, long term care, home or lab setting, etc.)
- Sample size
- Patient demographics (sex/gender (proportion), age, education (if present),
- Diagnostic group (if applicable; asymptomatic, mild, moderate, severe), comorbidity (if present, e.g. pre-existing neurocognitive disorders), other characteristics related to long COVID (i.e. impact of different variants of SARS-CoV-2, influence of COVID-19 vaccines or effects of reinfection)
- Symptom outcomes and time points or ranges (6, 12, 18, 24, or more, months)
- Outcome definition (including diagnostic criteria if available)
- Cognitive assessment methodology, measurement tools used, classification outcomes (with raw scores if included), rationale for including tests if stated
- Statistical approach used
- The authors' primary conclusions and, if available, explanations of the factors taken into consideration during the decision-making process

The relevant items were adapted from the Cochrane Handbook for Systematic Reviews of Interventions (24).

<u>Data analysis</u>

To facilitate data synthesis, all the studies in review will be synthesized by being charted by the reviewers, to document the data of interest for reviewed studies and to visualize in the final report.

For peer review only - http://bmjopen.bmj.com/site/about/guidelines.xhtml

In order to synthesize scoping evidence, both aggregative (summative) and configurative (organizing) synthesis approaches will be utilized, to comprehensively map the field. The data analysis should both reveal gaps in the literature, but also magnitudes of evidence, as such a mixed approach of both EGM and heat maps will be applied, along with other demographic visualizations as needed.

Specifically, EGM matrices will be populated to display presence or lack of evidence for cognitive outcomes, based on primary dimensions (duration, severity, tests used) and secondary dimensions (study design). This approach ensures plotting of heterogeneity in the field and gap identification. Heat map matrices will be used for various parameters depending on availability of data; e.g. long COVID label characterization (for duration, severity, study types), magnitude of symptoms reported per cognitive assessment and key impacting factors, with dimensions including SARS-CoV-2 impacting factors (influence of vaccines, variants, reinfections), comorbidities with respect to severity, duration and cognition reported, if sufficient data is present. Further layering of the matrix will be followed by group stratifications for identification of nuances.

This approach will support a quantifiable characterization of the concepts, which aids at giving insights into the field and objectives, given the sufficiency of collected data. Outcomes will be visualized in graphs and figures. The findings and figures will finally be disseminated in a narrative report.

<u>Quality Assessment</u>

In accordance with the guidelines developed by the Joanna Briggs Institute (JBI) for scoping reviews (25), no assessment of quality for included papers will be performed.

<u>Ethics and Dissemination</u>

The study does not directly involve human participants or personal data from potential participants, but only aggregated data from existing research. Therefore, no ethical approval is required. This protocol will enable for a scoping review whose results will be disseminated through its publication in a scientific journal, and within professional network.

For peer review only

For peer review only - http://bmjopen.bmj.com/site/about/guidelines.xhtml

Contributors: Conceiving the idea of the scoping review and protocol – SM. Design of the protocol – SM, MP. Search strategy – SM, CD, MP. Draft of the manuscript – SM. Review and final approval of the manuscript – MP.

Funding: This work is supported by the Luxembourg National Research Fund (FNR), Project n°20210613.

Ethics: Given the use of secondary data, no ethical approval will be required, as no participants will be involved in this research.

Peer review: External peer review

Competing interests: None declared

Implications: This scoping review aims to map out the present field of long COVID cognition by adding novel insights into the characteristics of persistent cognitive decline and investigating associated symptomatology and current testing conventions. By exploring the current scope of the field, valuable new insights add to insights of the nuanced dimensions of long COVID related cognitive decline and tailor healthcare interventions and rehabilitation programs accordingly.

Open access: This research was funded in whole by the Luxembourg National Research Fund (FNR), grant reference 16758430. For the purpose of open access, and in fulfilment of the obligations arising from the grant agreement, the author has applied a Creative Commons Attribution-ShareAlike 4.0 International (CC BY-SA 4.0) license to any Author Accepted Manuscript version arising from this submission. See: https://creativecommons.org/licenses/by-sa/4.0/deed.en

References

For peer review only - http://bmjopen.bmj.com/site/about/guidelines.xhtml

1. Post COVID-19 condition (Long COVID) [Internet]. [cited 2023 Dec 27]. Available from: https://www.who.int/europe/news-room/fact-sheets/item/post-covid-19-condition

2. Munblit D, O'Hara ME, Akrami A, Perego E, Olliaro P, Needham DM. Long COVID: aiming for a consensus. Lancet Respir Med. 2022 Jul;10(7):632–4.

3. Fernández-de-Las-Peñas C. Long COVID: current definition. Infection. 2022 Feb;50(1):285–6.

4. Thaweethai T, Jolley SE, Karlson EW, Levitan EB, Levy B, McComsey GA, et al. Development of a Definition of Postacute Sequelae of SARS-CoV-2 Infection. JAMA. 2023 Jun 13;329(22):1934–46.

5. Venkatesan P. NICE guideline on long COVID. Lancet Respir Med. 2021 Feb;9(2):129.

6. Shah W, Heightman M, O'Brien S. UK guidelines for managing long-term effects of COVID-19. Lancet. 2021 May 8;397(10286):1706.

7. Soriano JB, Murthy S, Marshall JC, Relan P, Diaz JV, WHO Clinical Case Definition Working Group on Post-COVID-19 Condition. A clinical case definition of post-COVID-19 condition by a Delphi consensus. Lancet Infect Dis. 2022 Apr;22(4):e102–7.

8. Sobrino-Relaño S, Balboa-Bandeira Y, Peña J, Ibarretxe-Bilbao N, Zubiaurre-Elorza L, Ojeda N. Neuropsychological deficits in patients with persistent COVID-19 symptoms: a systematic review and meta-analysis. Sci Rep. 2023 Jun 26;13(1):10309.

9. Nicotra A, Masserini F, Calcaterra F, Di Vito C, Doneddu PE, Pomati S, et al. What do we mean by long COVID? A scoping review of the cognitive sequelae of SARS-CoV-2 infection. Eur J Neurol. 2023 Dec;30(12):3968–78.

10. Hayes LD, Ingram J, Sculthorpe NF. More Than 100 Persistent Symptoms of SARS-CoV-2 (Long COVID): A Scoping Review. Front Med. 2021 Nov 1;8:750378.

11. Douaud G, Lee S, Alfaro-Almagro F, Arthofer C, Wang C, McCarthy P, et al. SARS-CoV-2 is associated with changes in brain structure in UK Biobank. Nature. 2022 Apr;604(7907):697–707.

12. Sampogna G, Di Vincenzo M, Giallonardo V, Perris F, Volpicelli A, Del Vecchio V, et al. The Psychiatric Consequences of Long-COVID: A Scoping Review. J Pers Med [Internet]. 2022 Oct 26;12(11). Available from: http://dx.doi.org/10.3390/jpm12111767

13. Hovagemyan F, Dugerdil A, Braggion A, Mallet L, Flahault A. Psychiatric consequences and issues of long COVID on patients with prior psychiatric comorbidities: a scoping review. Front Psychiatry. 2023 Jun 7;14:1181767.

14. Naji L, Dennis B, Morgan RL, Sanger N, Worster A, Paul J, et al. Investigating and addressing the immediate and long-term consequences of the COVID-19 pandemic on patients with substance use disorders: a scoping review and evidence map protocol. BMJ Open. 2021 Sep 1;11(9):e045946.

For peer review only

15. Díez-Cirarda M, Yus M, Gómez-Ruiz N, Polidura C, Gil-Martínez L, Delgado-Alonso C, et al. Multimodal neuroimaging in post-COVID syndrome and correlation with cognition. Brain. 2023 May 2;146(5):2142–52.

16. Ferrucci R, Dini M, Rosci C, Capozza A, Groppo E, Reitano MR, et al. One-year cognitive follow-up of COVID-19 hospitalized patients. Eur J Neurol. 2022 Jul;29(7):2006–14.

17. Arksey H, O'Malley L. Scoping studies: towards a methodological framework. Int J Soc Res Methodol. 2005 Feb 1;8(1):19–32.

18. Anderson S, Allen P, Peckham S, Goodwin N. Asking the right questions: scoping studies in the commissioning of research on the organisation and delivery of health services. Health Res Policy Syst. 2008 Jul 9;6:7.

19. Levac D, Colquhoun H, O'Brien KK. Scoping studies: advancing the methodology. Implement Sci. 2010 Sep 20;5:69.

20. Tricco AC, Lillie E, Zarin W, O'Brien KK, Colquhoun H, Levac D, et al. PRISMA Extension for Scoping Reviews (PRISMA-ScR): Checklist and Explanation. Ann Intern Med. 2018 Oct 2;169(7):467–73.

21. Page MJ, McKenzie JE, Bossuyt PM, Boutron I, Hoffmann TC, Mulrow CD, et al. The PRISMA 2020 statement: an updated guideline for reporting systematic reviews. Rev Esp Cardiol . 2021 Sep;74(9):790–9.

22. Schardt C, Adams MB, Owens T, Keitz S, Fontelo P. Utilization of the PICO framework to improve searching PubMed for clinical questions. BMC Med Inform Decis Mak. 2007 Jun 15;7:16.

23. Ouzzani M, Hammady H, Fedorowicz Z, Elmagarmid A. Rayyan—a web and mobile app for systematic reviews. Syst Rev. 2016 Dec 5;5(1):210.

24. Cochrane Collaboration. Cochrane Handbook for Systematic Reviews of Interventions. Cochrane Collaboration; 2021.

25. Peters MDJ, Godfrey CM, Khalil H, McInerney P, Parker D, Soares CB. Guidance for conducting systematic scoping reviews. Int J Evid Based Healthc. 2015 Sep;13(3):141–6.

For peer review only

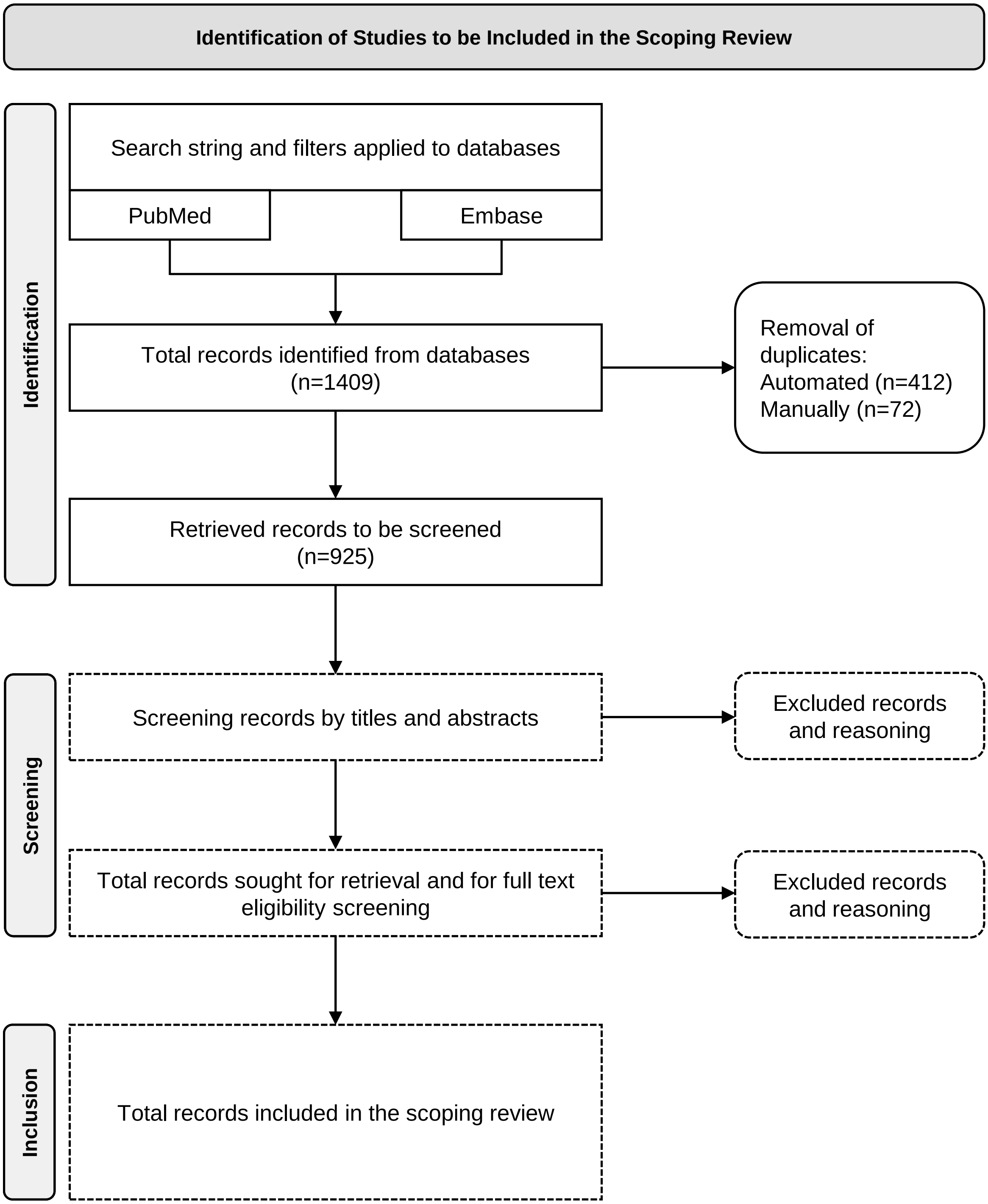

Figure 1: Flow Chart of the Screening Methodology in Concordance with the Reporting Items for Systematic Reviews and Meta-Analyses (PRISMA).

Reporting checklist for protocol of a systematic review and meta analysis.

Based on the PRISMA-P guidelines.

Moher D, Shamseer L, Clarke M, Ghersi D, Liberati A, Petticrew M, Shekelle P, Stewart LA. Preferred Reporting Items for Systematic Review and Meta-Analysis Protocols (PRISMA-P) 2015 statement. Syst Rev. 2015;4(1):1.

		Reporting Item	Page Number
**Title**			
Identification	#1a	Identify the report as a protocol of a systematic review	1
Update	#1b	If the protocol is for an update of a previous systematic review, identify as such	n/a
**Registration**			
	#2	If registered, provide the name of the registry (such as PROSPERO) and registration number	2
**Authors**			
Contact	#3a	Provide name, institutional affiliation, e-mail address of all protocol authors; provide physical mailing address of corresponding author	1
Contribution	#3b	Describe contributions of protocol authors and identify the guarantor of the review	13
**Amendments**			
	#4	If the protocol represents an amendment of a previously completed or published protocol, identify as such and list changes; otherwise, state plan for documenting important protocol amendments	n/a
**Support**			
Sources	#5a	Indicate sources of financial or other support for the review	13

For peer review only

Sponsor	#5b	Provide name for the review funder and / or sponsor	13
Role of sponsor or funder	#5c	Describe roles of funder(s), sponsor(s), and / or institution(s), if any, in developing the protocol	13
**Introduction**			
Rationale	#6	Describe the rationale for the review in the context of what is already known	3-4
Objectives	#7	Provide an explicit statement of the question(s) the review will address with reference to participants, interventions, comparators, and outcomes (PICO)	4
**Methods**			
Eligibility criteria	#8	Specify the study characteristics (such as PICO, study design, setting, time frame) and report characteristics (such as years considered, language, publication status) to be used as criteria for eligibility for the review	4-10
Information sources	#9	Describe all intended information sources (such as electronic databases, contact with study authors, trial registers or other grey literature sources) with planned dates of coverage	6-7
Search strategy	#10	Present draft of search strategy to be used for at least one electronic database, including planned limits, such that it could be repeated	6-10
Study records - data management	#11a	Describe the mechanism(s) that will be used to manage records and data throughout the review	10-12
Study records - selection process	#11b	State the process that will be used for selecting studies (such as two independent reviewers) through each phase of the review (that is, screening, eligibility and inclusion in meta-analysis)	7-8
Study records - data collection process	#11c	Describe planned method of extracting data from reports (such as piloting forms, done independently, in duplicate), any processes for obtaining and confirming data from investigators	10-12
Data items	#12	List and define all variables for which data will be sought (such as PICO items, funding sources), any pre-planned data assumptions and simplifications	10-11

For peer review only

Outcomes and prioritization	#13	List and define all outcomes for which data will be sought, including prioritization of main and additional outcomes, with rationale	10-11
Risk of bias in individual studies	#14	Describe anticipated methods for assessing risk of bias of individual studies, including whether this will be done at the outcome or study level, or both; state how this information will be used in data synthesis	11-12
Data synthesis	#15a	Describe criteria under which study data will be quantitatively synthesised	10-12
Data synthesis	#15b	If data are appropriate for quantitative synthesis, describe planned summary measures, methods of handling data and methods of combining data from studies, including any planned exploration of consistency (such as I2, Kendall's τ)	11-12
Data synthesis	#15c	Describe any proposed additional analyses (such as sensitivity or subgroup analyses, meta-regression)	11-12
Data synthesis	#15d	If quantitative synthesis is not appropriate, describe the type of summary planned	11-12
Meta-bias(es)	#16	Specify any planned assessment of meta-bias(es) (such as publication bias across studies, selective reporting within studies)	11-12
Confidence in cumulative evidence	#17	Describe how the strength of the body of evidence will be assessed (such as GRADE)	11-12

For peer review only

BMJ Open

Long COVID Cognitive Sequelae 6 Months Post-Infection and Beyond: A Scoping Review Protocol

Journal:	*BMJ Open*
Manuscript ID	bmjopen-2024-084798.R2
Article Type:	Protocol
Date Submitted by the Author:	13-May-2024
Complete List of Authors:	Monteiro, Sara; Luxembourg Institute of Health, Department of Precision Health<br>Dessenne, Coralie; Luxembourg Institute of Health, Science Office<br>Perquin, Magali ; Luxembourg Institute of Health,
**Primary Subject Heading**:	Public health
Secondary Subject Heading:	Mental health, Neurology, Infectious diseases, Research methods
Keywords:	Post-Acute COVID-19 Syndrome, Psychometrics, Chronic Disease, COVID-19, EPIDEMIOLOGY, MENTAL HEALTH

SCHOLARONE™
Manuscripts

Long COVID Cognitive Sequelae 6 Months Post Infection and Beyond:

A Scoping Review Protocol

Sara Monteiro[1,*], Coralie Dessenne[2], Magali Perquin[1].

[1] Department of Precision Health, Luxembourg Institute of Health, 1A-B, rue Thomas Edison, 1445 Strassen, Luxembourg

[2] Science Office, Luxembourg Institute of Health, 1A-B, rue Thomas Edison, 1445 Strassen, Luxembourg

***Corresponding Author:**

Sara Monteiro: sara.monteiro@lih.lu, ORCID: 0000-0001-9145-0063

Author Note:

Coralie Dessenne: coralie.dessenne@lih.lu, ORCID: 0000-0001-7723-0304

Magali Perquin: magali.perquin@lih.lu, ORCID: 0000-0002-0386-0212

Declaration: No conflict of interest to declare.

For peer review only

For peer review only - http://bmjopen.bmj.com/site/about/guidelines.xhtml

Abstract

Introduction: The novel and expanding field of long COVID research has undergone diverse methodological approaches in recent years. This protocol lays out the methodological approach, which aims at identifying nuances in current research. It underscores the necessity for a more precise understanding of prolonged cognitive sequelae and their relation to initial disease severity. The findings will add valuable insights for the development of targeted rehabilitation, healthcare interventions and thereby aid patients, clinicians, policymakers and researchers. Our upcoming research is hereby introduced.

Methods and Analysis: To map current research in the field, a scoping review will be conducted and documented in accordance with the Preferred Reporting Items for Systematic Reviews and Meta-Analyses - Scoping Review Extension standards. A systematic search of scientific databases (PubMed, EMBASE), presented 1409 eligible results, published up to 21 December 2023. After removal of duplicates, 925 articles were extracted for screening. Two independent reviewers will screen for titles, abstracts and full texts, to extract data, which will then be organized using charting software. Data for various variables, i.e. journal info, studied population demographics, study design, long COVID related data, cognitive outcomes and neuropsychological tests will be gathered. Descriptive analyses, Evidence Gap Maps (EGM), heat map quantifications and narrative synthesis will be conducted for reporting of results.

This scoping review has been registered with the Open Science Framework (https://doi.org/10.17605/OSF.IO/JHFX6).

Ethics and Dissemination: Ethical approval is not required, as the study does not involve human participants. The findings will be disseminated through a publication in a scientific journal and within the professional network.

Strengths and Limitations

- Cognitive symptoms will be characterized in detail, highlighting domains and particular sub-skills for in depth understanding of long COVID pathology.
- The multifaceted approach used (in-depth psychometrics, acute, and long-term COVID-19 symptomatology, as well as comorbid factors), enables a nuanced analysis and increases accuracy.
- A multidisciplinary team covering Information Science, Neuropsychology and Epidemiology add to the depth of interpretability and robustness of the review, by establishing a comprehensive search strategy and evaluation.
- Evidence maps will be used to address present heterogeneity in the field and thereby allowing for improved scientific understanding and enhanced clinical interventions.

Keywords: Long COVID, Post-Acute COVID-19 Syndrome, Cognition, Neuropsychology, Neuroepidemiology, Psychometrics

For peer review only - http://bmjopen.bmj.com/site/about/guidelines.xhtml

Background

According to the World Health Organization (WHO), 10–20% of individuals who contract SARS-CoV-2 infection may go on to experience chronic symptoms (1), that might persist from weeks and up to years (2). The overarching label for persistent symptoms is 'long COVID' (2,3); alternatively, 'post-acute sequelae of SARS-CoV-2 infection' (PASC) (4), 'Post COVID-19 Syndrome (PCS) (5,6) and 'Post COVID-19 Condition' (PCC) (7) have been proposed (2,3). Debates exploring consensus for a unified syndrome definition, labels and symptoms and their temporal evolution are currently ongoing. Establishing a unified model is challenging, particularly due to the varying severity, duration and overall heterogeneity of symptoms, the novelty of the field and a lack of unified assessment approaches used in studies (2,8), consequently also affecting the availability of healthcare management (3).

Attempts have been made to map out the characteristics of the post COVID-19 syndrome (9). Three core categories seem to dominate the clinical picture, namely cognitive, psychiatric and systemic issues (9). Consensus seems to exist for frequent symptoms of the systemic core, which includes fatigue, headaches, anosmia/ dysgeusia, myalgia, and respiratory difficulties, as well as the psychiatric core, mainly involving anxiety and depression (9,10). Memory, executive functioning and attentional deficits have been reported to be at the core of cognitive complaints, as well as cognitive slowing, commonly termed "brain fog" (9).

By dividing the condition into temporal stages, a number of models have been put forth during the last three years to explain and categorize the status of chronic symptoms (2,4). The majority of reviews and studies up to now focus on the early and acute phases following infection (6–10). Recent scoping reviews on COVID-19 reported cognitive symptoms and tests being used for screening up to six months maximum after the acute phase (9), as well as psychiatric conditions prior to and immediately after infection (11,12). Conversely, there has been a less comprehensive examination, especially regarding the neurocognitive symptom core, for long COVID symptoms beyond these immediate stages (8,9,13). However, a comparison between immediate and long-term effects has been reviewed, albeit on specific populations, such as patients with substance use disorders (14). Thus, although existing, to our knowledge, no study has addressed the specific and recurrent cognitive symptoms beyond 6 months after infection.

It is thus important to improve understanding of the current gap in the field relating to cognitive long-term sequelae, by identifying which symptoms are most commonly present in patients often referred to as "long haulers." Because long COVID has recently been found to be related with gray matter structural changes (13) and permanent alteration of brain connectivity, even eleven months after infection (15), it is essential to map the exact cognitive decline associated with the persistent manifestation (16). Attention, executive and memory components seem to lie at the core of the difficulties (9), however there is currently no single, cohesive concept about their persistency, which exact deficits are present to what extent and with regards to which initial severity, or on how to measure long COVID associated cognition (8,9). Additionally, it is

For peer review only

necessary to provide clarification regarding the heterogeneous use of study designs, assessment techniques, confounding caused by coexisting conditions that were not tested for, and a lack of comparison to earlier tests or controls (9). This approach will help with the optimization of existing medical diagnostic techniques, subsequent treatment plans and rehabilitative strategies and add to our understanding of the exact components of long COVID associated cognitive impairment.

Objectives:

The aim of this scoping review is to map out the persistent cognitive sequelae associated with long COVID symptomatology in adults, six months or more after infection.

- Characterize patterns of long COVID cognitive sequelae over time, specifically chronic symptoms present after at least six months or beyond.
- Investigate whether the initial disease severity (asymptomatic, mild, moderate, severe) influences the cognitive outcomes.
- Report on neuropsychological tests for cognitive assessment that were used to assess the cognitive changes, to understand the conceptual boundaries of current findings.
- Elucidate current gaps in the literature.

Methods and analysis:

Protocol and Registration

The protocol is registered under the Open Science Framework with OSF (https://doi.org/10.17605/OSF.IO/JHFX6).

Design

To guide the overall conception and reasoning, the Arksey and O'Malley methodological framework (17) will be applied to the design and decision making process of the systematic search. Additionally, the methodological concept proposed by the National Institute for Health Research (NIHR) Service Delivery and Organisation Research and Development Programme (18) will be used, with a main focus on 1) clarifying conceptual working definitions and to determine current boundaries of the field and concept, 2) identifying existing knowledge and present gaps, and 3) mapping existing evidence (19). Reporting the work will be aligned with the PRISMA Guidelines for Systematic Reviews and the Preferred Reporting Items for Systematic reviews and Meta-Analyses extension for scoping reviews (PRISMA-ScR) (20) and will follow the rationale of the Preferred Reporting Items for Systematic Reviews and Meta-Analyses (PRISMA) 2020 statement update (21), in the context of a scoping study.

For peer review only - http://bmjopen.bmj.com/site/about/guidelines.xhtml

To the best of our knowledge, after a preliminary search in November 2023 of PubMed, Google Scholar, OSF, PROSPERO, and the Cochrane Database of Systematic Reviews, no previous or upcoming reviews or protocols with a comparable design have been identified.

Patient and public involvement

No patients involved.

Eligibility Criteria

As shown in Table 1, the PICO Framework (22) served as a framework for determining the eligibility requirements.

Table 1: Eligibility Criteria Based on the PICO Framework.

Element	Inclusion criteria	Exclusion criteria
**P**articipants	Human participants<br><br>Articles involving adults ≥18 years old<br><br>Contains reliable information on previous COVID-19 infection<br><br>Chronic, recurrent, or onset of cognitive symptomatology ≥6 months after infection	Animal studies<br><br>Articles focusing on patients <18 years old
**I**ntervention or exposure of interest	No intervention reviewed; exposure is 1) SARS-CoV-2 initial infection, 2) cognitive testing after 6 months<br><br>Chronic, recurrent, or onset of cognitive symptomatology ≥6 months after infection<br><br>Preferred inclusion of studies reporting exclusion of people with pre-infection cognitive symptoms or disorders<br><br>Included studies report on tools used for identification of cognitive symptoms<br><br>At least 1 objective, validated and quantifiable cognitive assessment tool used	Articles only reporting outcomes for symptom duration <6 months<br><br>Articles which do not include cognitive symptoms

For peer review only

For peer review only - http://bmjopen.bmj.com/site/about/guidelines.xhtml

**C**omparator	The studies should include cognition outcomes long time after COVID-19<br><br>Different cognitive symptoms of interest<br><br>Different types of initial disease severity (which could have required different types of medical care, e.g. hospitalisation)<br><br>Different assessment modalities for cognitive performance	No information available about long COVID after 6 months or beyond<br><br>No initial infection to SARS-CoV-2
**O**utcome	Any neurocognitive outcome related to long COVID characteristics<br><br>“Long COVID” is not restricted to a specific definition or symptomatology, but exploration thereof remains open and will be explored<br><br>Alternative labels will be accepted<br><br>Any objective cognitive assessment modality will be included	Self-report of cognitive ability without objective measurement tool
Study design	Any type of study that assists in scoping the field, independent of the type, will be included (preference for longitudinal-, transversal-, with a cohort or a case-control-design, review, meta-analysis)	Studies with n<10 (e.g. case reports), qualitative studies
Article Type	Peer-reviewed articles<br><br>Available in full text	Grey literature, protocols, editorials, dissertations, book chapters, conference abstracts
Language	English manuscripts	
Study Period	From 2020	
Geography	No restriction	

For peer review only

<u>Information resources</u>

Articles will be extracted from the following databases:

- PubMed

- EMBASE

Search strategy

The search strategy was constructed together with a scientific librarian, to conduct a wide-ranging search within the selected databases, aiming at identification of an unbiased body of records. As outlined in Table 2, search terms from three themes ('long COVID', 'cognition' and 'assessment') will be conceptually arranged to be placed in comprehensive search strings for selection of relevant articles. Within a theme, search terms are arranged in conceptual clusters, which will be combined by syntax techniques such as Boolean operators ('OR', 'AND'), search tags (MeSH, title only) and filters ('humans', 'English language'). Additionally, word truncation (*) will be used to capture alternate word-endings. For example, the search term "COVID*" will capture instances of the word "COVID," and also include other labels or terms containing "COVID" within them (e.g. "long-COVID", "post-COVID", "COVID-19" etc.). Similarly, the search term "Visuo*" will include "Visuo-spatial", "Visuospatial", "Visuo-constructive", etc.

The precise search strings generated from this technique were applied to each database and will be documented and reported in terms of the PRISMA-ScR Framework (20) in the final review article.

Primary Search

A primary search conducted on December 21, 2023, resulted in the identification of 1409 articles. After an automated duplicate screening, 997 remained and were added to EndNote 21 to manage references and produce a bibliography for future screening. 925 records remained for screening after manual duplicate deletion, as depicted in Figure 1.

Screening and Study Selection

The review will be conducted in six stages: the problem is identified and the study design is being developed, the search strategy is established and implemented, literature screening, data collection, data analysis, write-up and dissemination of findings.

Following the identification of articles matching the search strategy and duplicate removal, references in their totality will be documented and managed with the review software Rayyan.ai (23). Articles will be systematically screened for eligibility in a stepwise manner by the reviewers. The number or studies after each step and exclusion decisions will be recorded, as illustrated in Graph 1.

1) Eligibility by title and abstract: Two independent reviewers will be screening for titles and abstracts of the article, to identify eligibility for inclusion. The screening decisions will be compared between the two reviewers. A third independent reviewer will be consulted for any remaining discrepancies.

For peer review only - http://bmjopen.bmj.com/site/about/guidelines.xhtml

2) Full text screening: The reviewer pair will assess the article selection for their full texts. Articles will be excluded if 1) data of interest is not reported, 2) patient population not eligible, 3) non-preferred study design, 4) non-preferred article type, or any other non-alignment with the eligibility criteria. Discrepancies will be solved as described in the previous section.

Figure 1: Flow Diagram of the Study Selection Methodology in Concordance with the Reporting Items for Systematic Reviews and Meta-Analyses (PRISMA 2020) (21).

Table 2: **Conceptual Search Strategy and Search Terms.**
The Theme column specifies the specific topics of focus. The Key Concept column outlines the different notions covered by each corresponding Theme. The Search Terms/Strings column provides:

- The terms in the article either in any field, either in a specific field such as the Title.
- The exact standardized terms to index articles in the databases - they are assigned to articles to categorize their content in a consistent and organized manner. These standardized terms are known as MeSH terms (Medical Subject Headings) in MEDLINE/PubMed and EMTREE terms in EMBASE.

Theme	Key Concept		Search Terms/ Strings in PubMed	Search Terms/ Strings in EMBASE
**Long COVID**	**COVID**	**#1**	"COVID*"[TI] OR "SARS-CoV-2"[TI]	'covid*':ti OR 'sars-cov-2':ti
	**Infection**	**#2**	"COVID-19"[Mesh] OR "SARS-CoV-2"[Mesh]	'coronavirus disease 2019'/exp
	**Persistence**	**#3**	"long" OR "persistent" OR "chronic" OR "post-acute" OR "sequela*"	'long' OR 'persistent' OR 'chronic' OR 'post-acute' OR 'sequela*'
	**Long COVID**	**#4**	"Post-Acute COVID-19 Syndrome"[Mesh]	'long covid'/exp
**Cognition**	**Memory**	**#5**	"memor*"[TI]	'memor*':ti
	**Attention**	**#6**	"attention"[TI]	'attention':ti

For peer review only - http://bmjopen.bmj.com/site/about/guidelines.xhtml

	**Executive Functioning**	**#7**	"executive function*"[TI]	'executive function*'
	**Vision**	**#8**	"visual" OR "visuo*"[TI]	'visual':ti OR 'visuo*':ti
	**Language**	**#9**	"language"[TI] OR "verbal"[TI] OR "fluency"[TI] OR "speech"[TI]	'language':ti OR 'verbal':ti OR 'fluency':ti OR 'speech':ti
	**Cognition**	**#10**	"cogniti*"[TI]	'cogniti*':ti
	**Mental State**	**#11**	"brain fog"[TI] OR "confusion"[TI] OR "orientation"[TI] OR "disorientation"[TI]	'brain fog':ti OR 'confusion':ti OR 'orientation':ti OR 'disorientation':ti
**Assessment**	**Paradigms**	**#12**	"neuropsycholog*"[TI] OR "neurocogniti*"[TI]	'neuropsycholog*':ti OR 'neurocogniti*':ti
	**Testbatteries**	**#13**	"MMSE" OR "MoCA" OR "CERAD" OR "TMT" OR "SDMT" OR "symbol digit modalities test" OR "Stroop" OR "N-BACK" OR "GO-NO*" OR "STMB*	'mmse' OR 'moca' OR 'cerad' OR 'tmt' OR 'sdmt' OR 'symbol digit modalities test' OR 'stroop' OR 'n-back' OR 'go-no*' OR 'stmb*'

The final strategy combined successively all these search step as follows:

(((#1 OR #2) AND #3) OR #4) AND (#5 OR #6 OR #7 OR #8 OR #9 OR #10 OR #11 OR #12 OR #13)

<u>Data management (included and excluded papers)</u>

To maximize and document the objectivity of the screening process, the decisions and justifications for inclusion or exclusion will be documented in a digitalized spreadsheet table. Reference management, bibliography generation and write-up will be assisted by usage of EndNote 21, the screening will be conducted with Rayyan.ai (23) and a digital spread sheet will be used for charting the data.

For peer review only

Data collection process and extraction (Charting)

Rayyan.ai (23) and a digital spreadsheet will be utilized to extract and chart the data among the reviewer team members. All included papers will be reviewed by two reviewers, each independently charting data for the following variables:

- Author(s)
- Year of publication
- Title
- DOI
- Study objective
- Study design (e.g. cohort, multi-center studies, cross-sectional, longitudinal etc.)
- Country of study
- Setting context (e.g. acute, primary health care, long term care, home or lab setting, etc.)
- Sample size
- Patient demographics (sex/gender (proportion), age, education (if present),
- Diagnostic group (if applicable; asymptomatic, mild, moderate, severe), comorbidity (if present, e.g. pre-existing neurocognitive disorders), other characteristics related to long COVID (i.e. impact of different variants of SARS-CoV-2, influence of COVID-19 vaccines or effects of reinfection)
- Symptom outcomes and time points or ranges (6, 12, 18, 24, or more, months)
- Outcome definition (including diagnostic criteria if available)
- Cognitive assessment methodology, measurement tools used, classification outcomes (with raw scores if included), rationale for including tests if stated
- Statistical approach used
- The authors' primary conclusions and, if available, explanations of the factors taken into consideration during the decision-making process

For peer review only

For peer review only - http://bmjopen.bmj.com/site/about/guidelines.xhtml

The relevant items were adapted from the Cochrane Handbook for Systematic Reviews of Interventions (24).

Data analysis

To facilitate data synthesis, all the studies in review will be synthesized by being charted by the reviewers, to document the data of interest for reviewed studies and to visualize in the final report. In order to synthesize scoping evidence, both aggregative (summative) and configurative (organizing) synthesis approaches will be utilized, to comprehensively map the field. The data analysis should both reveal gaps in the literature, but also magnitudes of evidence, as such a mixed approach of both EGM and heat maps will be applied, along with other demographic visualizations as needed.

Specifically, EGM matrices will be populated to display presence or lack of evidence for cognitive outcomes, based on primary dimensions (duration, severity, tests used) and secondary dimensions (study design). This approach ensures plotting of heterogeneity in the field and gap identification, which aids at identifying research density. Heat map matrices will be used for various parameters depending on availability of data; e.g. long COVID label characterization (for duration, severity, study types), magnitude of symptoms reported per cognitive assessment and key impacting factors, with dimensions including SARS-CoV-2 impacting factors (influence of vaccines, variants, reinfections), comorbidities with respect to severity, duration and cognition reported, if sufficient data is present. Further layering of the matrix will be followed by group stratifications for identification of nuances.

This approach will support a quantifiable characterization of the concepts, which aids at exploring the field and objectives, given the sufficiency of collected data. Outcomes will be visualized in graphs and figures. The findings and figures will finally be disseminated in a narrative report, complementing the analysis by integrating findings across different methodologies and outcomes and compare them to existing models and theories.

Quality Assessment

In accordance with the guidelines developed by the Joanna Briggs Institute (JBI) for scoping reviews (25), no assessment of quality for included papers will be performed.

Ethics and Dissemination

The study does not directly involve human participants or personal data from potential participants, but only aggregated data from existing research. Therefore, no ethical approval is required. This protocol will enable for a scoping review whose results will be disseminated through its publication in a scientific journal, and within professional network.

For peer review only - http://bmjopen.bmj.com/site/about/guidelines.xhtml

Contributors: Conceiving the idea of the scoping review and protocol – SM. Design of the protocol – SM, MP. Search strategy – SM, CD, MP. Draft of the manuscript – SM. Review and final approval of the manuscript – MP.

Funding: This work is supported by the Luxembourg National Research Fund (FNR), Project n°20210613.

Ethics: Given the use of secondary data, no ethical approval will be required, as no participants will be involved in this research.

Peer review: External peer review

Competing interests: None declared

Implications: This scoping review aims to map out the present field of long COVID cognition by adding novel insights into the characteristics of persistent cognitive decline and investigating associated symptomatology and current testing conventions. By exploring the current scope of the field, valuable new insights add to insights of the nuanced dimensions of long COVID related cognitive decline and tailor healthcare interventions and rehabilitation programs accordingly.

Open access: This research was funded in whole by the Luxembourg National Research Fund (FNR), grant reference 16758430. For the purpose of open access, and in fulfilment of the obligations arising from the grant agreement, the author has applied a Creative Commons Attribution-ShareAlike 4.0 International (CC BY-SA 4.0) license to any Author Accepted Manuscript version arising from this submission. See: https://creativecommons.org/licenses/by-sa/4.0/deed.en

For peer review only - http://bmjopen.bmj.com/site/about/guidelines.xhtml

References

1. Post COVID-19 condition (Long COVID) [Internet]. [cited 2023 Dec 27]. Available from: https://www.who.int/europe/news-room/fact-sheets/item/post-covid-19-condition

2. Munblit D, O'Hara ME, Akrami A, Perego E, Olliaro P, Needham DM. Long COVID: aiming for a consensus. Lancet Respir Med. 2022 Jul;10(7):632–4.

3. Fernández-de-Las-Peñas C. Long COVID: current definition. Infection. 2022 Feb;50(1):285–6.

4. Thaweethai T, Jolley SE, Karlson EW, Levitan EB, Levy B, McComsey GA, et al. Development of a Definition of Postacute Sequelae of SARS-CoV-2 Infection. JAMA. 2023 Jun 13;329(22):1934–46.

5. Venkatesan P. NICE guideline on long COVID. Lancet Respir Med. 2021 Feb;9(2):129.

6. Shah W, Heightman M, O'Brien S. UK guidelines for managing long-term effects of COVID-19. Lancet. 2021 May 8;397(10286):1706.

7. Soriano JB, Murthy S, Marshall JC, Relan P, Diaz JV, WHO Clinical Case Definition Working Group on Post-COVID-19 Condition. A clinical case definition of post-COVID-19 condition by a Delphi consensus. Lancet Infect Dis. 2022 Apr;22(4):e102–7.

8. Sobrino-Relaño S, Balboa-Bandeira Y, Peña J, Ibarretxe-Bilbao N, Zubiaurre-Elorza L, Ojeda N. Neuropsychological deficits in patients with persistent COVID-19 symptoms: a systematic review and meta-analysis. Sci Rep. 2023 Jun 26;13(1):10309.

9. Nicotra A, Masserini F, Calcaterra F, Di Vito C, Doneddu PE, Pomati S, et al. What do we mean by long COVID? A scoping review of the cognitive sequelae of SARS-CoV-2 infection. Eur J Neurol. 2023 Dec;30(12):3968–78.

10. Hayes LD, Ingram J, Sculthorpe NF. More Than 100 Persistent Symptoms of SARS-CoV-2 (Long COVID): A Scoping Review. Front Med. 2021 Nov 1;8:750378.

11. Sampogna G, Di Vincenzo M, Giallonardo V, Perris F, Volpicelli A, Del Vecchio V, et al. The Psychiatric Consequences of Long-COVID: A Scoping Review. J Pers Med [Internet]. 2022 Oct 26;12(11). Available from: http://dx.doi.org/10.3390/jpm12111767

12. Hovagemyan F, Dugerdil A, Braggion A, Mallet L, Flahault A. Psychiatric consequences and issues of long COVID on patients with prior psychiatric comorbidities: a scoping review. Front Psychiatry. 2023 Jun 7;14:1181767.

13. Douaud G, Lee S, Alfaro-Almagro F, Arthofer C, Wang C, McCarthy P, et al. SARS-CoV-2 is associated with changes in brain structure in UK Biobank. Nature. 2022 Apr;604(7907):697–707.

14. Naji L, Dennis B, Morgan RL, Sanger N, Worster A, Paul J, et al. Investigating and addressing the immediate and long-term consequences of the COVID-19 pandemic on

For peer review only

For peer review only - http://bmjopen.bmj.com/site/about/guidelines.xhtml

patients with substance use disorders: a scoping review and evidence map protocol. BMJ Open. 2021 Sep 1;11(9):e045946.

15. Díez-Cirarda M, Yus M, Gómez-Ruiz N, Polidura C, Gil-Martínez L, Delgado-Alonso C, et al. Multimodal neuroimaging in post-COVID syndrome and correlation with cognition. Brain. 2023 May 2;146(5):2142–52.

16. Ferrucci R, Dini M, Rosci C, Capozza A, Groppo E, Reitano MR, et al. One-year cognitive follow-up of COVID-19 hospitalized patients. Eur J Neurol. 2022 Jul;29(7):2006–14.

17. Arksey H, O'Malley L. Scoping studies: towards a methodological framework. Int J Soc Res Methodol. 2005 Feb 1;8(1):19–32.

18. Anderson S, Allen P, Peckham S, Goodwin N. Asking the right questions: scoping studies in the commissioning of research on the organisation and delivery of health services. Health Res Policy Syst. 2008 Jul 9;6:7.

19. Levac D, Colquhoun H, O'Brien KK. Scoping studies: advancing the methodology. Implement Sci. 2010 Sep 20;5:69.

20. Tricco AC, Lillie E, Zarin W, O'Brien KK, Colquhoun H, Levac D, et al. PRISMA Extension for Scoping Reviews (PRISMA-ScR): Checklist and Explanation. Ann Intern Med. 2018 Oct 2;169(7):467–73.

21. Page MJ, McKenzie JE, Bossuyt PM, Boutron I, Hoffmann TC, Mulrow CD, et al. The PRISMA 2020 statement: an updated guideline for reporting systematic reviews. Rev Esp Cardiol . 2021 Sep;74(9):790–9.

22. Schardt C, Adams MB, Owens T, Keitz S, Fontelo P. Utilization of the PICO framework to improve searching PubMed for clinical questions. BMC Med Inform Decis Mak. 2007 Jun 15;7:16.

23. Ouzzani M, Hammady H, Fedorowicz Z, Elmagarmid A. Rayyan—a web and mobile app for systematic reviews. Syst Rev. 2016 Dec 5;5(1):210.

24. Cochrane Collaboration. Cochrane Handbook for Systematic Reviews of Interventions. Cochrane Collaboration; 2021.

25. Peters MDJ, Godfrey CM, Khalil H, McInerney P, Parker D, Soares CB. Guidance for conducting systematic scoping reviews. Int J Evid Based Healthc. 2015 Sep;13(3):141–6.

For peer review only

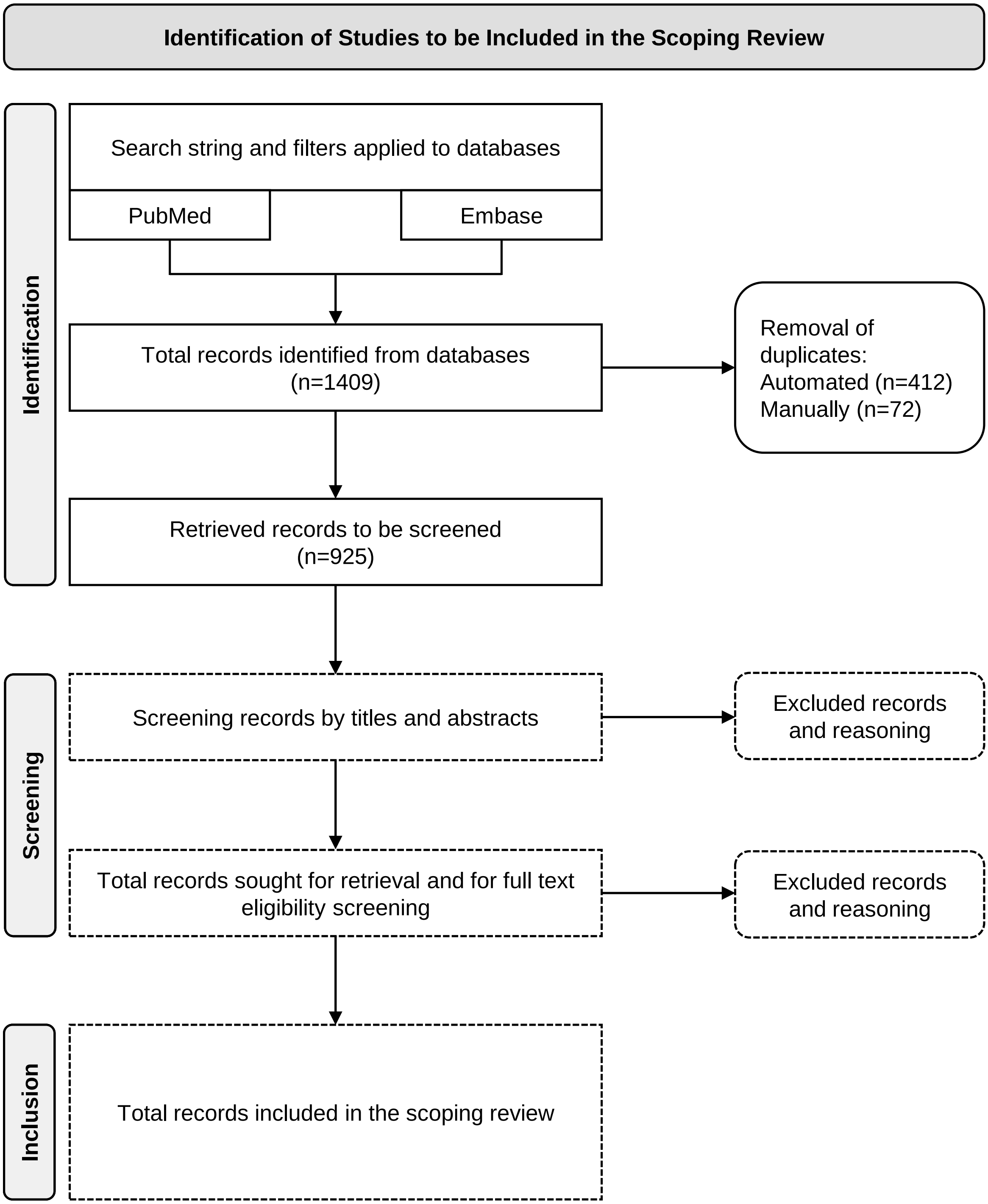

Figure 1: Flow Chart of the Screening Methodology in Concordance with the Reporting Items for Systematic Reviews and Meta-Analyses (PRISMA).

Reporting checklist for protocol of a systematic review and meta analysis.

Based on the PRISMA-P guidelines.

Moher D, Shamseer L, Clarke M, Ghersi D, Liberati A, Petticrew M, Shekelle P, Stewart LA. Preferred Reporting Items for Systematic Review and Meta-Analysis Protocols (PRISMA-P) 2015 statement. Syst Rev. 2015;4(1):1.

		Reporting Item	Page Number
**Title**			
Identification	#1a	Identify the report as a protocol of a systematic review	1
Update	#1b	If the protocol is for an update of a previous systematic review, identify as such	n/a
**Registration**			
	#2	If registered, provide the name of the registry (such as PROSPERO) and registration number	2
**Authors**			
Contact	#3a	Provide name, institutional affiliation, e-mail address of all protocol authors; provide physical mailing address of corresponding author	1
Contribution	#3b	Describe contributions of protocol authors and identify the guarantor of the review	13
**Amendments**			
	#4	If the protocol represents an amendment of a previously completed or published protocol, identify as such and list changes; otherwise, state plan for documenting important protocol amendments	n/a
**Support**			
Sources	#5a	Indicate sources of financial or other support for the review	13

For peer review only

Sponsor	#5b	Provide name for the review funder and / or sponsor	13
Role of sponsor or funder	#5c	Describe roles of funder(s), sponsor(s), and / or institution(s), if any, in developing the protocol	13
**Introduction**			
Rationale	#6	Describe the rationale for the review in the context of what is already known	3-4
Objectives	#7	Provide an explicit statement of the question(s) the review will address with reference to participants, interventions, comparators, and outcomes (PICO)	4
**Methods**			
Eligibility criteria	#8	Specify the study characteristics (such as PICO, study design, setting, time frame) and report characteristics (such as years considered, language, publication status) to be used as criteria for eligibility for the review	4-10
Information sources	#9	Describe all intended information sources (such as electronic databases, contact with study authors, trial registers or other grey literature sources) with planned dates of coverage	6-7
Search strategy	#10	Present draft of search strategy to be used for at least one electronic database, including planned limits, such that it could be repeated	6-10
Study records - data management	#11a	Describe the mechanism(s) that will be used to manage records and data throughout the review	10-12
Study records - selection process	#11b	State the process that will be used for selecting studies (such as two independent reviewers) through each phase of the review (that is, screening, eligibility and inclusion in meta-analysis)	7-8
Study records - data collection process	#11c	Describe planned method of extracting data from reports (such as piloting forms, done independently, in duplicate), any processes for obtaining and confirming data from investigators	10-12
Data items	#12	List and define all variables for which data will be sought (such as PICO items, funding sources), any pre-planned data assumptions and simplifications	10-11

For peer review only

Outcomes and prioritization	#13	List and define all outcomes for which data will be sought, including prioritization of main and additional outcomes, with rationale	10-11
Risk of bias in individual studies	#14	Describe anticipated methods for assessing risk of bias of individual studies, including whether this will be done at the outcome or study level, or both; state how this information will be used in data synthesis	11-12
Data synthesis	#15a	Describe criteria under which study data will be quantitatively synthesised	10-12
Data synthesis	#15b	If data are appropriate for quantitative synthesis, describe planned summary measures, methods of handling data and methods of combining data from studies, including any planned exploration of consistency (such as I2, Kendall's τ)	11-12
Data synthesis	#15c	Describe any proposed additional analyses (such as sensitivity or subgroup analyses, meta-regression)	11-12
Data synthesis	#15d	If quantitative synthesis is not appropriate, describe the type of summary planned	11-12
Meta-bias(es)	#16	Specify any planned assessment of meta-bias(es) (such as publication bias across studies, selective reporting within studies)	11-12
Confidence in cumulative evidence	#17	Describe how the strength of the body of evidence will be assessed (such as GRADE)	11-12

For peer review only
